# TreC: A Critical Mediator of lysoPC‐Induced Hypervirulence in *Streptococcus suis*

**DOI:** 10.1155/tbed/8378013

**Published:** 2026-04-18

**Authors:** Xuefeng Cao, Jiajia Zheng, Song Xue, Qinyu Wei, Marc M. S. M. Wösten, Lianci Peng, Zhiwei Li, Rendong Fang

**Affiliations:** ^1^ Joint International Research Laboratory of Animal Health and Animal Food Safety, College of Veterinary Medicine, Southwest University, Chongqing, China, swu.edu.cn; ^2^ Department Biomolecular Health Sciences, Utrecht University, Utrecht, Netherlands, uu.nl

**Keywords:** lysophosphatidylcholine, metabolic adaptations, *Streptococcus suis*, TreC, virulence enhancement

## Abstract

*Streptococcus suis*, a major zoonotic pathogen, employs diverse strategies to enhance virulence, yet the role of host‐derived metabolites in its pathogenicity remains underexplored. Here, we reveal a novel mechanism by which *S. suis* utilizes lysophosphatidylcholine (lysoPC), a host lipid signaling molecule, to enhance its virulence through *treC*‐dependent carbohydrate metabolic reprogramming. Transcriptomic analysis of lysoPC‐treated *S. suis* revealed significant upregulation of carbohydrate metabolism genes, particularly *treC*, which encodes trehalose‐6‐phosphate hydrolase (TreC) and drives trehalose biosynthesis by catalyzing the conversion of trehalose‐6‐phosphate to trehalose, a pathway absent in mammals. In murine infection models, lysoPC‐exposed wild‐type (WT) *S. suis* caused more rapid mortality, increased bacterial loads in systemic organs, and enhanced cytotoxicity towards human endothelial cells. Strikingly, these effects were abolished in Δ*treC* mutants but restored upon genetic complementation, confirming *treC* as indispensable for lysoPC‐mediated virulence. Notably, transcriptomic analysis revealed that canonical virulence genes remained unaltered, underscoring metabolic adaptation as the primary driver. Histopathological analysis further demonstrated lysoPC‐enhanced pulmonary and hepatic damage in a *treC*‐dependent manner. Our findings establish that *S. suis* exploits environmental lysoPC to rewire central metabolism via *treC*, bypassing traditional virulence pathways. This metabolic‐virulence coupling highlights *treC* as a therapeutic target to disrupt lysoPC‐driven pathogenicity. By bridging host lipid signaling with bacterial metabolic plasticity, this study advances our understanding of niche‐specific virulence strategies and offers new avenues for combating *S. suis* infections.

## 1. Introduction

*Streptococcus suis*, a notorious Gram‐positive zoonotic pathogen, continues to challenge global health and swine industries, causing life‐threatening infections such as meningitis, septicemia, and pneumonia [1, 2]. Despite its clinical importance, the molecular mechanisms underlying its virulence remain incompletely understood. During colonization and infection, *S. suis* encounters various host niches, such as saliva, the tonsils, the airway epithelium, the intestinal epithelium, the genital tract, joints, blood, or cerebrospinal fluid (CSF). The metabolic adaptation to these different body parts is accompanied by variation of metabolic gene expression and enables *S. suis* to utilize host‐derived nutrients and signaling molecules, thereby supporting survival and proliferation under stress conditions and indirectly enhancing pathogenicity [3]. Recent studies have highlighted the role of host‐derived metabolites in modulating bacterial pathogenicity. For instance, iron often serves as limiting factors, prompting bacteria to activate specialized uptake systems that enhance survival and virulence, bile acids and short‐chain fatty acids in the gastrointestinal tract have been shown to influence bacterial stress responses and metabolic pathways, affecting pathogenic potential [4–6]. These findings suggest that pathogens may exploit host molecules to enhance their survival and virulence in hostile environments. Although classical virulence factors like suilysin (SLY) and muramidase‐released protein (MRP) are extensively characterized, emerging evidence suggests that the pathogenic potential of *S. suis* may be influenced by its ability to adapt to host‐derived metabolic signals rather than relying solely on traditional virulence factors [7, 8]. Lysophospholipids (LPLs) are bioactive signaling molecules containing a single fatty acid tail. In eukaryotic cells, LPLs exhibit diverse biological properties, such as promoting cell growth or acting as potent lipid mediators [8]. Previously, we have identified that *Campylobacter jejuni* utilizes LPLs to defend itself against the toxic components of bile and to optimally adapt to the intestinal niche [6, 9]. Lysophosphatidylcholine (lysoPC), a major LPL found in animal tissues and body fluids, with concentrations of 200–300 μM, has emerged as a critical regulator of bacterial behavior [10, 11]. lysoPC is not only involved in eukaryotic cell signaling processes such as proliferation, apoptosis, and inflammation, but also plays a role in bacterial pathogenesis. However, the specific mechanisms by which lysoPC influences bacterial virulence remain poorly characterized.

Previous research has shown that host lysoPC trigger the release of the proinflammatory flagellin and directly promotes the expression of *Salmonella* invasion‐related genes, thereby enhancing the innate and inflammatory responses toward this bacterium [12–14]. These studies highlight lysoPC’s role in modulating virulence; however, its involvement in metabolic adaptation, distinct from traditional virulence gene activation, remains a significant gap in our understanding.

In this study, we elucidated a novel mechanism by which *S. suis* hijacks lysoPC from the host environment to enhance its virulence through rewiring its central carbohydrate metabolism via TreC, which drives trehalose biosynthesis, bypassing the activation of traditional virulence factors. These insights offer therapeutic avenues for targeting *treC* or its pathways, potentially disrupting lysoPC’s virulence‐enhancing effects in *S. suis* infections.

## 2. Materials and Methods

### 2.1. Bacteria and Mammalian Cell Culture

The *S. suis* wild‐type (WT) virulence strain P1/7 (serotype 2) originally isolated from a pig dying of meningitis [15], was routinely grown at 37°C in Todd Hewitt broth (THB; BBL Microbiology Systems, Cockeysville, MA, USA) as described [16]. Bacterial concentrations were quantified by a colony counting assay, and bacteria were diluted with cell culture medium to the indicated concentrations for experimental use. Human cerebral microvascular endothelial cells (hCMEC/D3) cells (JNO‐H0520, Jennio Biotech) were grown in 25 cm^2^ flasks in Dulbecco’s modified Eagle’s medium (DMEM) containing 10% fetal calf serum (FCS).

### 2.2. Mutagenesis of the *treC* and *lacD* Genes

To disrupt the *treC* and *lacD* genes, the upstream (L) and downstream (R) homologous arms flanking each target locus (*treC* or *lacD*) were PCR‐amplified from *S. suis* P1/7 genomic DNA using the primer pairs lacD‐L‐F/lacD‐L‐R, lacD‐R‐F/lacD‐R‐R, and treC‐L‐F/treC‐L‐R, treC‐R‐F/treC‐R‐R, respectively (Supporting Information 1: Table S1). The L and R fragments were fused by overlap‐extension PCR using the primer pairs lacD‐L‐F/lacD‐R‐R and treC‐L‐F/treC‐R‐R, respectively (Supporting Information 1: Table S1), and cloned into the temperature‐sensitive shuttle vector pSET4S [17] containing the spectinomycin resistance gene, generating pSET4S‐Δ*treC* and pSET4S‐Δ*lacD*. The constructs were verified by sequencing and subsequently used to mutate *S. suis* P1/7 WT using electroporation [18]. Transformants were first selected at the permissive temperature (28°C) on spectinomycin (100 µg/mL). Spectinomycin‐resistant clones were shifted to the non‐permissive temperature (37°C) in antibiotic‐free medium to allow allelic exchange. Candidate colonies were then replica‐plated onto THB containing spectinomycin and antibiotic‐free THB, respectively. After being incubated at 37°C for 16 h, clones that failed to grow on spectinomycin but grew on nonselective THB were scored as double‐crossover mutants. Gene deletions were confirmed by PCR. The mutants and parent strain showed similar bacterial growth rates in THB (data not shown).

### 2.3. Construction of the *treC* and *lacD* Complementation Plasmids

To complement the *treC* and *lacD* mutants, the *treC* and *lacD* genes containing homologous arms identical to the end sequence of linear pset2 plasmid were amplified from the chromosomal DNA of *S. suis* P1/7 with the primers treC‐F/treC‐R and lacD‐F/lacD‐R (Supporting Information 1: Table S1). The products were ligated into the shuttle plasmid pset2, respectively [19]. The constructs were verified by sequencing and subsequently used to complement Δ*treC* and Δ*lacD* using electroporation.

### 2.4. Mice

WT C57BL/6 mice were purchased from the Chongqing Academy of Chinese Material Medical (Chongqing, China). All the mice were maintained in specific pathogen‐free (SPF) conditions until they were used for experiments at 8–10 weeks of age. All the animal experiments were approved by the Institutional Animal Care and Use Committee (IACUC) of Southwest University, Chongqing, China (IACUC‐20210215‐05). All procedures were performed in accordance with relevant guidelines and regulations.

### 2.5. Murine Infection Assay

Commercial lysoPC was purchased from Avanti Polar Lipids Inc. (Alabama, USA). Female WT mice (*n* = 6 in each group) were intraperitoneally injected with lysoPC (10 µM)‐treated or untreated *S. suis* P1/7, its isogenic *treC* deletion mutant (Δ*treC*), *lacD* mutant (Δ*lacD*), or the corresponding complemented strains Δ*treC*+*treC*, Δ*lacD*+*lacD*. After lysoPC pretreatment, bacterial cells were collected by centrifugation, washed three times with sterile DPBS to remove residual lysoPC, and resuspended in fresh DPBS prior to injection. The blank control group received DPBS or lysoPC injections. The survival rates of mice were monitored over a 2‐day period. Survival of the mice was recorded every 6 h postchallenge. Mice that were still alive after 1 week were euthanized. For tissue analysis, female WT mice (*n* = 6 in each group) were intraperitoneally injected with lysoPC (10 µM)‐treated or untreated *S. suis* WT strain P1/7 or the Δ*treC* mutant, while the blank control group was injected with DPBS. At 24 h postinfection, 500 μL of blood was collected from the mice via the ophthalmic plexus and transferred to a heparin sodium anticoagulant tube. Mice were then sacrificed under anesthesia with ether inhalation, and liver/spleen/lung/kidney/brain tissues (weighing 0.1 ± 0.01 g) were aseptically removed and placed in a preweighed 2 mL grinding tube containing 1 mL ice bath DPBS. After grinding, 100 μL of each of the above tissue samples were serially diluted in DPBS and applied onto THB agar plates. Plates were incubated at 37°C until a single colony appeared. Bacterial load assessment was determined by colony counting.

### 2.6. *S. suis* Tissue Culture Infection Assay

*S. suis* P1/7 is able to adhere to hCMEC/D3 cells but not invade them [20], therefore, these cells were used to quantify *S. suis* adhesion. Cells seeded in 48‐well plates were infected with lysoPC‐pretreated (2 h, 10 µM) or untreated *S. suis* at a multiplicity of infection (MOI) of 10. At 5 h postinfection, cells were washed three times with sterile PBS to remove nonadherent bacteria. Cell lysis was achieved using 0.1% Triton X‐100 for 10 min at room temperature. Following serial dilution of the samples, bacteria were plated, and the number of adherent bacteria was determined by colony counting after incubation.

### 2.7. RNA‐Seq

*S. suis* P1/7 cultures were diluted to an OD_600_ of 0.05 in THB and grown for 5 h with or without 10 µM lysoPC at 37°C. RNA was extracted from *S. suis* using the RNA‐Bee kit (Tel‐Test). RNA samples were treated with RNAse‐free DNase I (Invitrogen) according to the manufacturer’s manual. RNA‐seq analysis was performed as previously described [21].

### 2.8. Hemolysis and Cytotoxicity Assays

Hemolysis and cytotoxicity were determined as described [9] using lysoPC pretreated or untreated bacteria. Hemolysis was expressed as a percentage of cell lysis (absorbance OD_420_) compared to the positive control (cells lysed with milli‐Q water). Host cell cytotoxicity was determined by measurement of the lactate dehydrogenase (LDH) release from 10^6^ tissue culture cells at 5 h after addition of *S. suis* at a bacterium to host cell ratio of 100:1.

### 2.9. *Propidium Iodide* (*PI*) *Staining*

hCMEC/D3 cells were prepared in 48‐well plates and infected with *S. suis* P1/7 as described above. After infection, the cells were stained with Hoechst/PI (KeyGEN BioTECH, Jiangsu, China). Following staining, cells were observed by fluorescence microscopy (Olympus, Tokyo, Japan) with excitation/emission wavelengths set at 535/617 nm.

### 2.10. Western Blot Analysis

Western blot analysis was performed as previously described [22]. hCMEC/D3 were prepared in 12‐well plates and infected with *S. suis* P1/7 as described above. The supernatants were subsequently collected at 2, 4 and 6 h posttreatment and concentrated with 20% (w/v) trichloroacetate (TCA), after which the cells were lysed with 1 × SDS loading buffer (Beyotime, Beijing, China). Next, the supernatants and cell lysates were subjected to 12% SDS‒PAGE and then transferred to polyvinylidene difluoride (PVDF) membranes by electroblotting. The membranes were blocked with 5% nonfat dry milk and then immunoblotted with the indicated antibodies (Abs), including anti‐ZO1 (1:5000, Beyotime, Beijing, China), anti‐occludin (1:5000, Beyotime, Beijing, China), and anti‐β‐actin (1:1000, Beyotime, Beijing, China).

### 2.11. Histopathological Analysis

Lungs, livers, kidneys, and brains were harvested 24 h after infection as described above, fixed in 10% formaldehyde, dehydrated, and embedded in paraffin. Hematoxylin and eosin (H&E) staining was performed to evaluate histopathological alterations. A veterinary pathologist examined the stained sections and scored them in a blinded manner using a previously described method [23, 24]. Briefly, the following scoring system was used: 0 (−), regular range; 1 (+), changes occurring that barely exceed those within the normal range (i.e., minimal changes); 2 (++), the extent of diseased tissue ranges from 11% to 20% of the examined tissue; 3 (+++), the extent of diseased tissue ranges from 21% to 40% of the examined tissue; 4 (++++), the extent of diseased tissue ranges from 41% to 100% of the examined tissue.

### 2.12. Statistical Analysis

The data presented are the means ± SEM derived from three independent experiments for each experimental group. Statistical significance between groups was assessed using one‐way and two‐way ANOVA. The levels of significance are denoted as follows:  ^∗^ *p* ≤ 0.05,  ^∗∗^ *p* ≤ 0.01,  ^∗∗∗^ *p* ≤ 0.001, and  ^∗∗∗∗^ *p* ≤ 0.0001, with “ns” indicating no statistical significance. These analyses allow for rigorous evaluation of the experimental data, ensuring robust and reliable interpretations of the results.

## 3. Results

### 3.1. lysoPC Enhances *S. suis* Virulence in Cellular and Murine Models

To investigate whether lysoPC, a lipid *S. suis* is unable to synthesize itself [8], plays a role in *S. suis* pathogenicity, we first examined its effects on host cell interactions by assessing its cytotoxicity in hCMEC/D3. Pretreatment of *S. suis* with 2–20 µM equivalents of lysoPC (composition: 69% lysoPC 16:0; 24.6% lysoPC 18:0; 3.4% lysoPC 18:1; 1.4% lysoPC 16:1; 0.3% lysoPC 14:0; 0.3% lysoPC 18:2; 1% unidentified species) for 2 h induced up to 5.7‐fold increase in host cell death compared to untreated bacteria detected by the LDH release assay (Figure 1A). Extending the pretreatment duration to 5 h prior to infection under the same conditions further enhanced virulence, with up to an 8.3‐fold increase. This cytotoxicity was specific for lysoPC, as other LPLs species, including lysophosphatidylethanolamine (lysoPE), lysophosphatidylinositol (lysoPI), lysophosphatidic acid (lysoPA), lysophosphatidylglycerol (lysoPG), and lysophosphatidylserine (lysoPS) had no effect on the LDH release (Supporting Information 7: Figure S1A). The cytotoxic effect was concentration‐dependent (Figure 1A), suggesting that lysoPC primes *S. suis* for enhanced host cell damage. To further confirm that the observed cytotoxicity was attributable to bacterial priming rather than to lysoPC itself, host cells were treated with 2–20 μM lysoPC alone for 5 h. No concentration‐dependent cytotoxicity was detected under these conditions (Supporting Information 7: Figure S1B), indicating that the increased cytotoxicity was not caused by residual lysoPC. Cell death analysis by PI staining showed a consistent trend, with increased red fluorescence indicating membrane‐damaged cells, that lysoPC‐treated (10 µM, 5 h) *S. suis* demonstrated a robust cytotoxicity to host cells (Supporting Information 8: Figure S2). In parallel, pretreatment of *S. suis* with 10 µM lysoPC increased adherence to hCMEC/D3 monolayers by 12‐fold (Figure 1B). Furthermore, lysoPC triggered disruption of endothelial tight junctions, as evidenced by a significant reduction in zonula occludens‐1 (ZO‐1) protein levels in hCMEC/D3 monolayers from 2 to 6 h postinfection (Figure 1C). To assess the in vivo relevance, we next assessed its effect on virulence in murine infection models. Intraperitoneal challenge of mice with *S. suis* pretreated 5 h with 10 µM lysoPC resulted in significantly enhanced mortality compared to untreated bacteria (Figure 1D). In line with this, lysoPC‐exposed *S. suis* exhibited a 1.2‐fold higher bacterial load in blood and 1.5‐, 1.2‐, 1.2‐ and 1.2‐fold increases in brain, lung, renal, and liver colonization, respectively, at 24 h postinfection (Figure 1E). These findings establish that lysoPC enhances *S. suis* cytotoxicity, promotes epithelial adhesion, and compromises host barrier integrity through ZO‐1 degradation, collectively facilitating systemic invasion.

Figure 1lysoPC enhance *S. suis* virulence. (A) LDH release of hCMEC/D3 cells infected with *S. suis* pretreated with different concentration of lysoPC. (B) Adhesion assay of *S. suis* to hCMEC/D3 cells after 5 h infection. (C) Western blot analysis of ZO‐1, occludin, and β‐actin expression in hCMEC/D3 cells infected with lysoPC‐treated or untreated *S. suis*. (D) Survival assays of mice after *S. suis*, lysoPC, lysoPC‐pretreated *S. suis* or DPBS (negative control) injection. (E) Bacterial load in blood, brain, kidney, lung, and liver of mice after lysoPC‐treated or untreated *S. suis* infection. Data of three independent experiments with three independent preparations of bacterial samples are presented as mean values ± standard deviation,  ^∗^ *p*  < 0.1,  ^∗∗^ *p*  < 0.01,  ^∗∗∗^ *p*  < 0.001,  ^∗∗∗∗^ *p*  < 0.0001, ns *p*  > 0.1.

**Figure:**
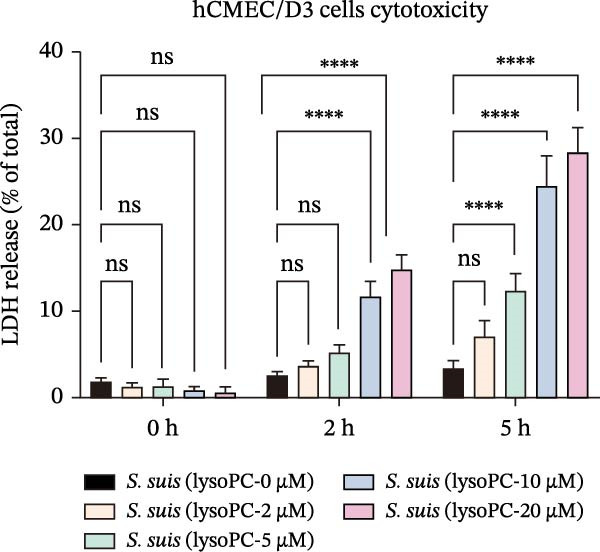
(A)

**Figure:**
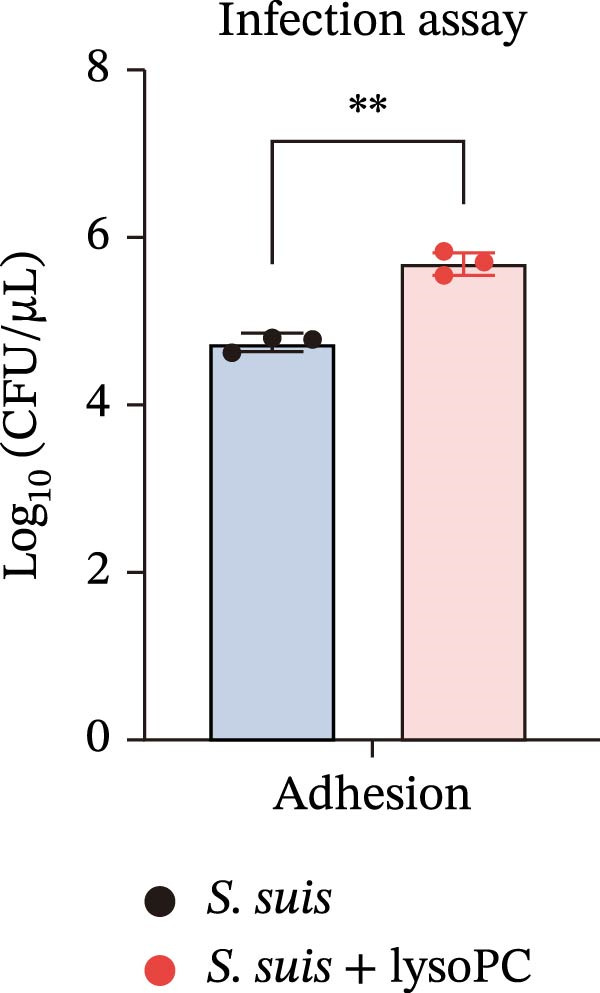
(B)

**Figure:**
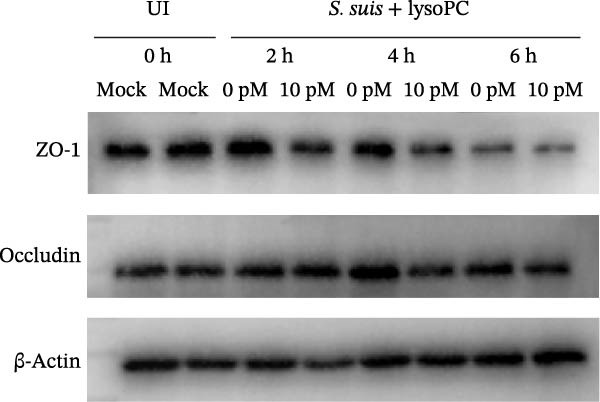
(C)

**Figure:**
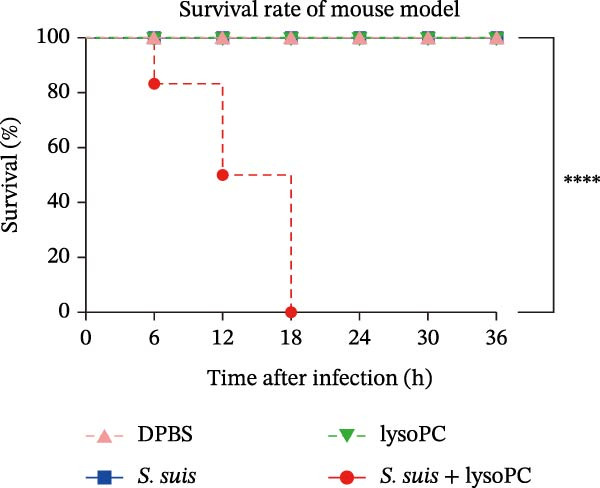
(D)

**Figure:**
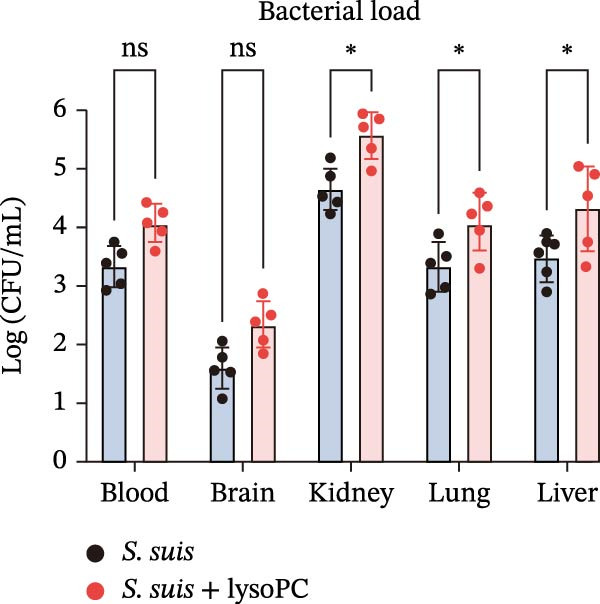
(E)

### 3.2. lysoPC Selectively Activates *S. suis* Carbohydrate Metabolism Without Inducing Canonical Virulence Genes

To better understand how lysoPC can promote *S. suis* virulence, we performed RNA‐seq on the WT grown for 5 h in THB with or without lysoPC. Here, the RNA‐seq results have been analyzed using the formula (*S. suis* WT − *S. suis* WT lysoPC), with a positive value indicating that lysoPC increases the transcription of the gene (upregulation), whereas a negative value indicates a reduction in transcription (downregulation). Hierarchical clustering analysis revealed distinct global gene expression profiles between lysoPC‐treated and untreated *S. suis*, indicating a pronounced transcriptomic response to lysoPC exposure (Supporting Information 2: Table S2, Supporting Information 9: Figure S3). The separation of samples into discrete clusters based on treatment condition suggests that lysoPC induces a coordinated regulatory shift in bacterial gene expression. Comparative transcriptomic profiling of *S. suis* treated with 10 µM lysoPC versus untreated controls identified 256 differentially expressed genes (Supporting Information 3: Table S3A), with 73 upregulated (including 26 significantly upregulated, fold change > 3, Supporting Information 3: Table S3B) and 183 downregulated loci (Figure 2A). Real‐time PCR of 11 highly regulated genes yielded no gross differences in results between the real‐time PCR and RNA‐seq data (Supporting Information 4: Table S4A,B), verifying the RNA‐seq results. Notably, canonical virulence genes (e.g., *sly*, *mrp*, and *fbps*) remained transcriptionally unaltered, indicating that lysoPC enhances *S. suis* pathogenicity through metabolic adaptations rather than direct activation of classical virulence factors. The 26 most strongly upregulated genes were predominantly associated with energy carbohydrate metabolism pathways, key genes included *lac* (*lacA*, *lacB*, *lacD*, *lacT*, *lacF*, *lacE*, and *lacG*) and *tre* (*treC* and *treP*) family genes. Further KEGG pathway analysis and Gene Ontology (GO) terms associated *tre* family genes with the trehalose biosynthesis pathway and *lac* family genes with galactose catabolism via the tagatose‐6‐phosphate pathway (Supporting Information 5: Table S5, Supporting Information 6: Table S6, Supporting Information 10: Figure S4, Supporting Information 11: Figure S5). RNA‐seq data identified *treC* and *lacD* as the most significantly upregulated genes within the trehalose and galactose metabolism pathways, respectively (Figure 2B). To elucidate the mechanism by which lysoPC enhances *S. suis* virulence, we prioritized *treC* and *lacD* as candidate genes based on transcriptomic and functional enrichment analyses. Particularly, neither *treC* nor *lacD* is associated with classical virulence regulons, reinforcing the hypothesis that lysoPC enhances pathogenicity through metabolic rewiring rather than direct activation of canonical virulence factors. To validate their roles, we generated Δ*treC* and Δ*lacD* deletion mutants and assessed their virulence attenuation in subsequent mechanistic studies.

Figure 2The transcriptional influence of lysoPC on *S. suis*. (A) Volcano plot was created by all differentially expressed genes. *Y*‐axis shows the mean expression value of log_10_ (*p* value), and the *x*‐axis displays the log_2_‐fold change value. (B) The heatmap and hierarchical clustering of the highly regulated genes are presented. The RNA‐seq experiments were repeated three times with similar results.

**Figure:**
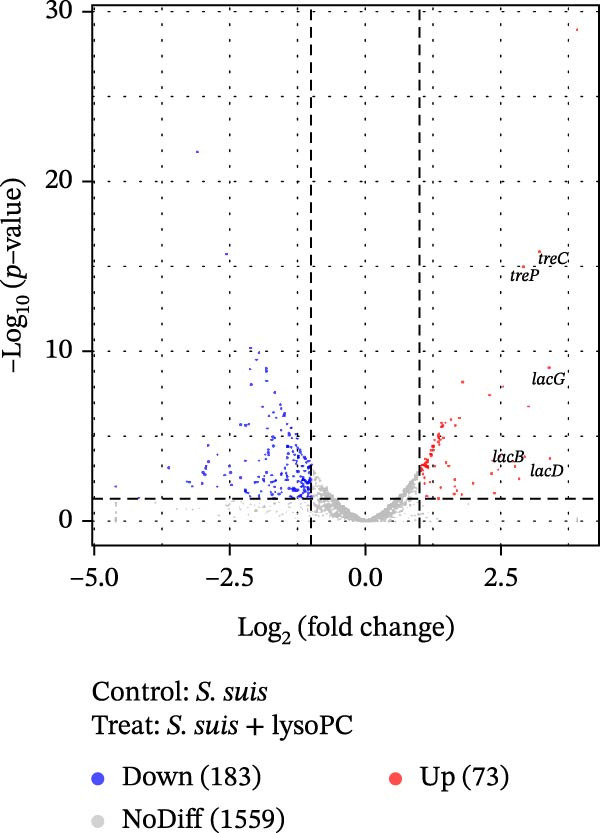
(A)

**Figure:**
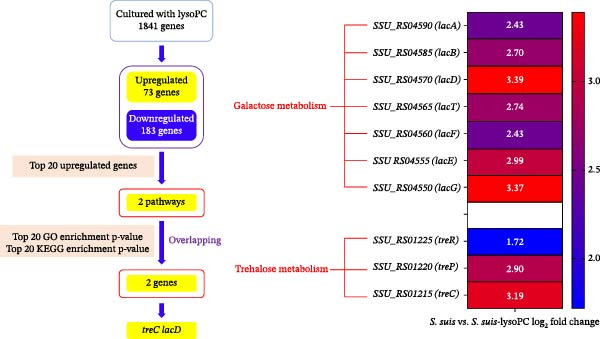
(B)

### 3.3. lysoPC Enhances *S. suis* Virulence in a *treC*‐Dependent Manner

Transcriptomic profiling revealed that lysoPC enhances *S. suis* virulence by reprogramming galactose and trehalose metabolism, with *treC* and *lacD* emerging as the most upregulated genes in these pathways. To dissect the mechanistic roles of *treC* and *lacD* in lysoPC‐driven virulence, we evaluated cytotoxicity by membrane integrity and in vivo lethality, using isogenic mutants and complemented strains.

lysoPC‐treated WT *S. suis* exhibited a 2.3‐fold increase in LDH release from hCMEC/D3 cells compared to untreated WT (*p* < 0.0001; Figure 3A), indicating enhanced cytotoxicity. Similarly, lysoPC partially boosted cytotoxicity in Δ*lacD*, elevating LDH release by 3.7‐fold relative to untreated Δ*lacD* (*p* < 0.0001; Figure 3A). In contrast, lysoPC had no effect on Δ*treC* cytotoxicity (1.5‐fold vs. untreated Δ*treC*; *p* = 0.12; Figure 3A). Genetic complementation of Δ*treC* fully restored lysoPC‐driven cytotoxicity, with Δ*treC*+*treC* achieving LDH levels equivalent to Δ*lacD* + lysoPC (2.0‐ and 2.6‐fold increases, respectively; *p*  < 0.0001; Figure 3A). These results suggest that TreC is the key factor for lysoPC‐induced *S. suis* cytotoxicity. To exclude general growth defects as the cause of attenuated virulence, 10 µM lysoPC was added to THB to evaluate its effect on in vitro bacterial growth. Growth curves revealed no significant differences among WT *S. suis* and the Δ*lacD* and Δ*treC* mutants (Supporting Information 13: Figure S7), indicating that lysoPC specifically primes virulence rather than enhancing overall bacterial fitness. To further investigate the role of TreC and LacD in the pathogenesis of *S. suis*, murine survival assays were performed. We found that lysoPC significantly enhanced *S. suis* virulence, as evidenced by 100% mortality in mice infected with lysoPC‐treated WT *S. suis* by 24 h (median survival: 18 h; *p* < 0.0001 vs. untreated WT), whereas lysoPC failed to boost virulence in the Δ*treC* mutant (16.6% mortality at 48h; Figure 3B). In contrast, lysoPC‐treated Δ*lacD* exhibited partial virulence restoration, reducing survival to 50% at 24 h, though remaining attenuated compared to lysoPC‐treated WT *S. suis*. Genetic complementation of Δ*lacD* and Δ*treC* restored virulence to WT levels in murine infection models (*lacD*: 100% mortality at 24 h, median survival: 18 h; Δ*treC*: 100% mortality at 18 h, median survival: 15 h; Supporting Information 12: Figure S6). These experimental findings demonstrate that TreC serves as the central mediator of lysoPC‐induced *S. suis* virulence enhancement.

Figure 3TreC as a key regulator of lysoPC‐induced *S. suis* virulence. (A) LDH release of hCMEC/D3 cells infected with *S. suis*, Δ*treC*, Δ*treC* + *treC*, Δ*lacD*, Δ*lacD* + *lacD*, lysoPC‐pretreated *S. suis*, lysoPC‐pretreated Δ*treC*, lysoPC‐pretreated Δ*lacD*, lysoPC‐pretreated Δ*lacD* + *lacD*, and lysoPC‐pretreated Δ*treC*+*treC* strains. (B) Survival assays of mice after *S. suis*, Δ*treC*, Δ*lacD*, lysoPC‐pretreated *S. suis*, lysoPC‐pretreated Δ*treC*, lysoPC‐pretreated Δ*lacD* or DPBS (negative control) injection. (C–G) Bacterial load in lung, blood, liver, kidney, and brain of mice after *S. suis*, Δ*treC*, lysoPC‐pretreated *S. suis*, lysoPC‐pretreated Δ*treC* infection. Data of three independent experiments with three independent preparations of bacterial samples are presented as mean values ± standard deviation,  ^∗^ *p*  < 0.1,  ^∗∗^ *p*  < 0.01,  ^∗∗∗^ *p*  < 0.001,  ^∗∗∗∗^ *p*  < 0.0001, ns *p*  > 0.1.

**Figure:**
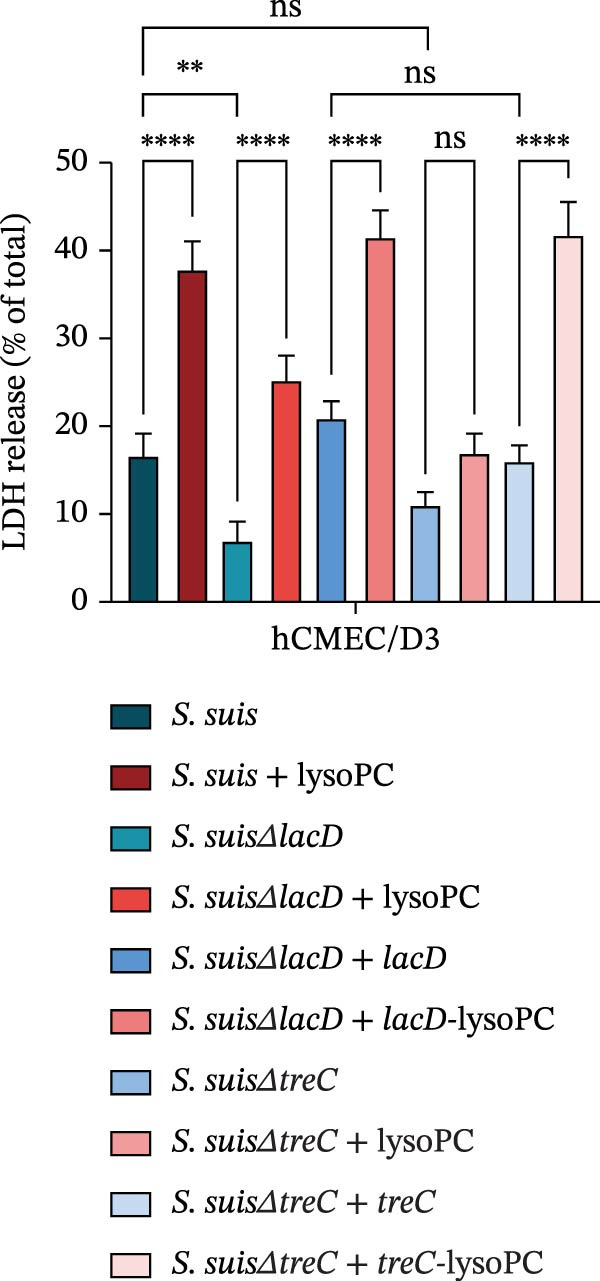
(A)

**Figure:**
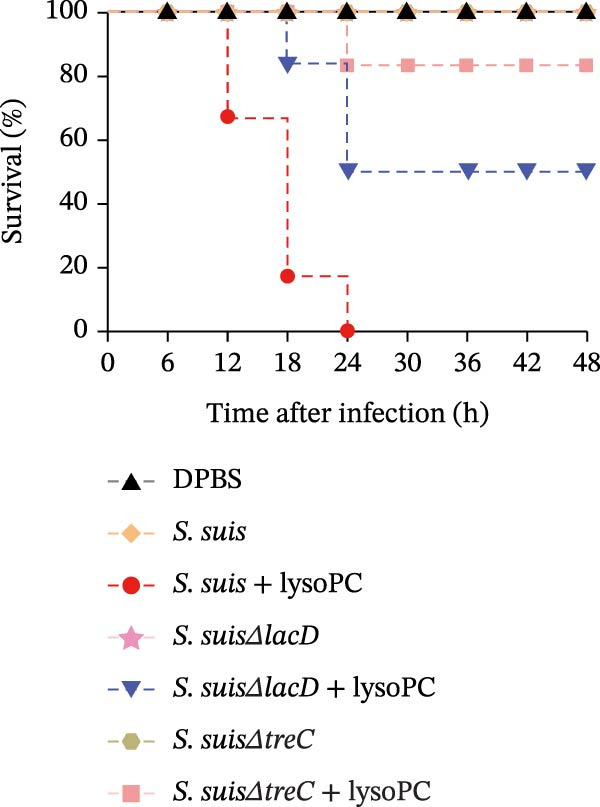
(B)

**Figure:**
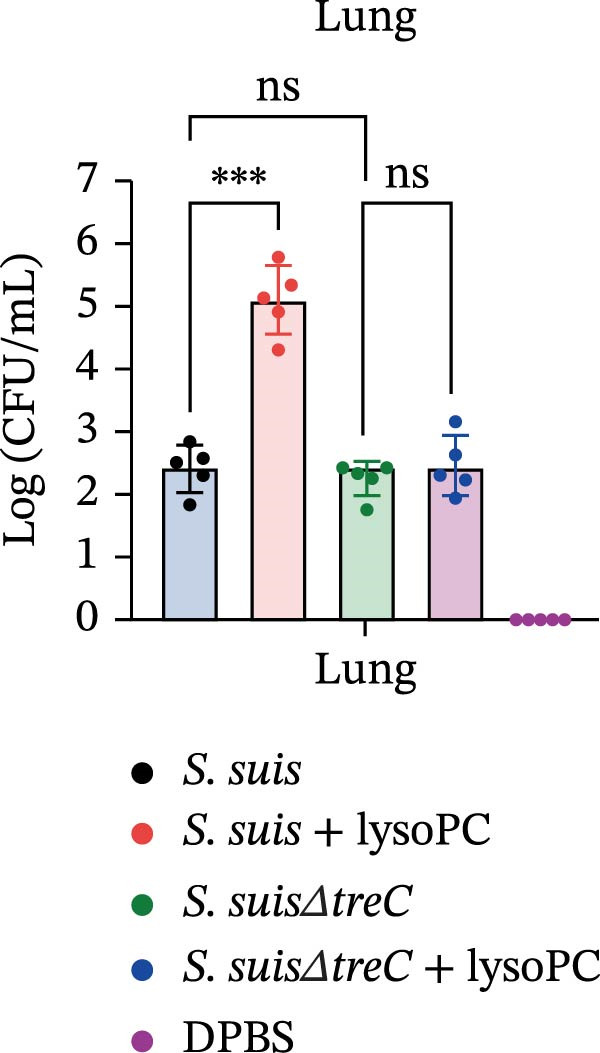
(C)

**Figure:**
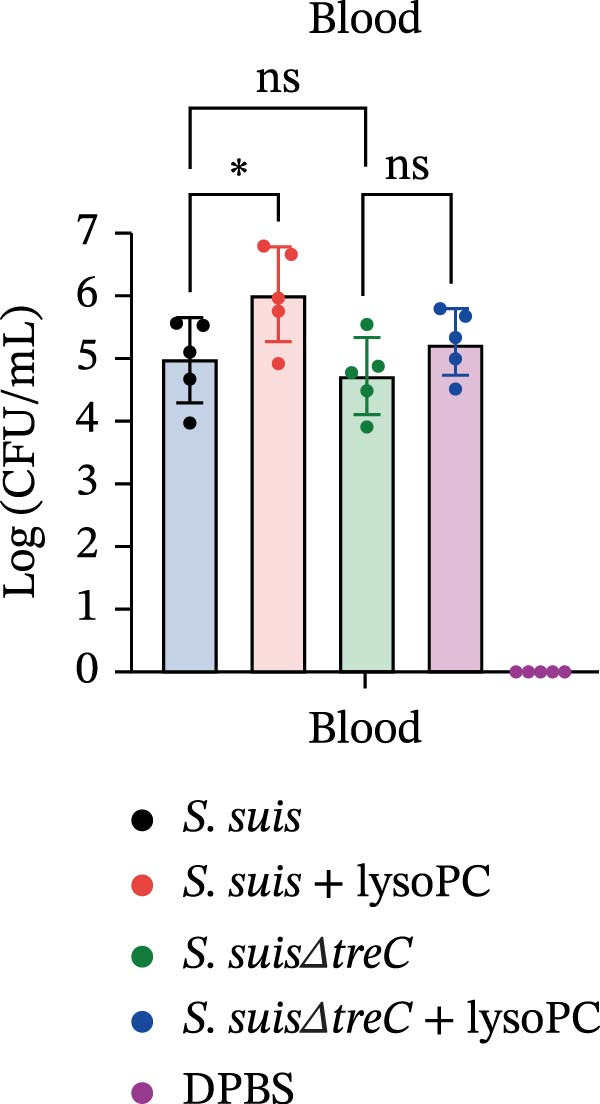
(D)

**Figure:**
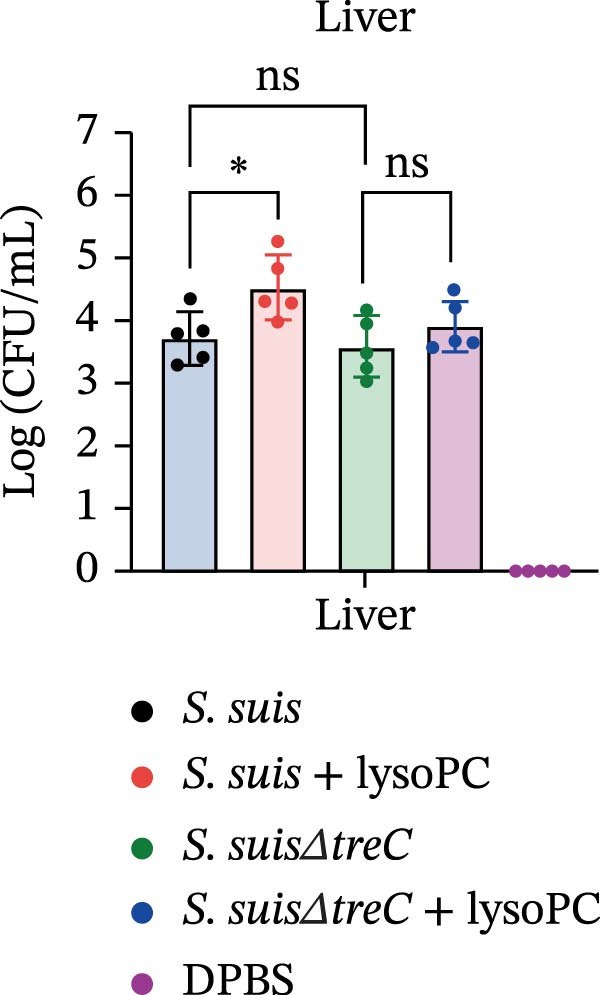
(E)

**Figure:**
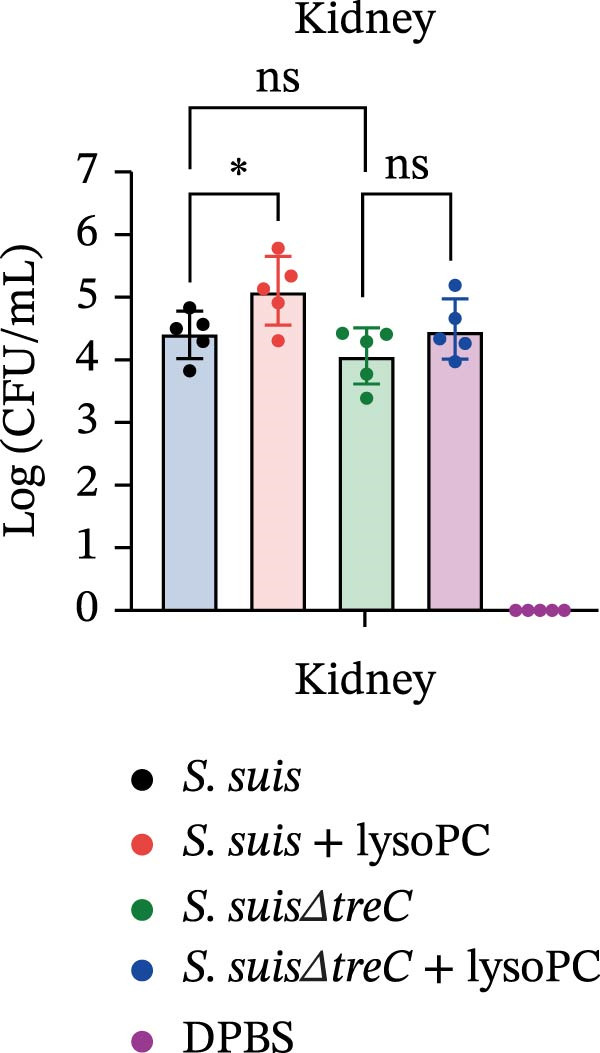
(F)

**Figure:**
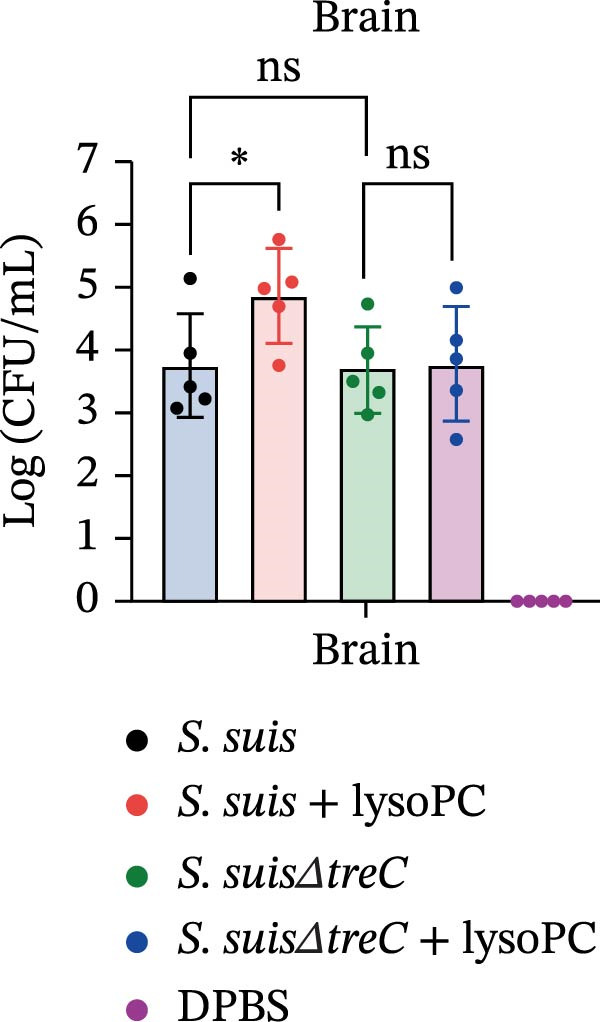
(G)

lysoPC‐treated WT *S. suis* exhibited significantly higher bacterial burdens compared to untreated WT in all organs analyzed at 24 h postinfection. The log lung CFU counts increased by 2.1‐fold (*p*  < 0.0001; Figure 3C), while blood, liver, kidney, and brain colonization have also remarkably increased (*p*  < 0.001; Figure 3C–G). In contrast, lysoPC failed to enhance the virulence of the Δ*treC* mutant. Bacterial loads in Δ*treC* + lysoPC‐infected mice remained comparable to untreated Δ*treC* (lung: *p* = 0.95; blood: *p* = 0.7; liver: *p* = 0.69; kidney: *p* = 0.72; brain: *p* = 1.0). These systemic bacterial loads correspond well with survival outcomes. Notably, untreated Δ*treC* and WT displayed comparable baseline colonization, revealing that *treC* is dispensable for basal virulence but essential for lysoPC‐driven enhancement. Cell death analysis by PI staining confirmed these findings: lysoPC‐treated *S. suis* caused an increase in PI‐positive cells compared to untreated *S. suis* (Supporting Information 14: Figure S8), indicating significant cytotoxicity. In contrast, lysoPC had no effect on Δ*treC* cytotoxicity, with PI staining levels indistinguishable from untreated Δ*treC* (Supporting Information 14: Figure S8). Together these results show that pathogenicity of *S. suis* strongly increases in a TreC‐dependent manner after incubation with lysoPC.

### 3.4. Histopathological Analysis of lysoPC‐Induced Virulence Modulation in *S. suis* Infection

To assess the impact of lysoPC in modulating *S. suis* virulence, histopathological evaluations were performed in the lungs, liver, kidney, and brain of mice following bacterial challenge. Among the organs assessed, lysoPC exhibited the most pronounced effects on pulmonary and hepatic pathology, with distinct histological differences observed across experimental groups.

In lung tissues, the WT + lysoPC group demonstrated pronounced pathological alterations, characterized by alveolar epithelial hyperplasia graded as “++” according to the standardized four‐grade lesion scoring system [25], indicating involvement of 11%–20% of the examined tissue (Figure 4A and Supporting Information 15: Figure S9). This change was significantly attenuated in the Δ*treC* group, Δ*treC* + lysoPC group, and WT control group, which exhibited only minimal alveolar epithelial hyperplasia, graded as “+,” which was barely above the normal range. The WT, Δ*treC*, WT + lysoPC, and Δ*treC* + lysoPC groups also displayed mild perivascular and perilobular inflammatory infiltrates, without evidence of alveolar structural disruption.

Figure 4TreC deletion blocks lysoPC‐induced systemic organ damage caused by *S. suis*. Histopathological changes in (A) lung and (B) liver of *S. suis*, Δ*treC*, lysoPC‐pretreated *S. suis*, lysoPC‐pretreated Δ*treC* infection (magnification, 400×). Red square frames mark the hyperplastic alveolar epithelial cells; yellow square frames mark the proliferative fibrous tissues; green square frames mark the punctate necrosis of hepatocytes.

**Figure:**
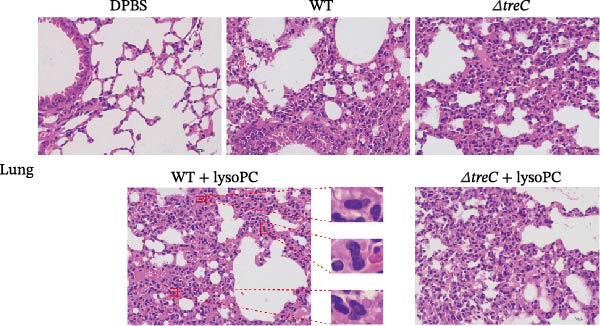
(A)

**Figure:**
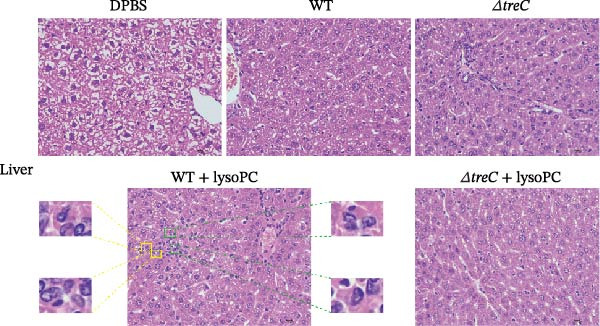
(B)

In hepatic tissues, lysoPC treatment exacerbated *S. suis*‐induced pathology in a genotype‐dependent manner. The WT + lysoPC group exhibited the most severe lesions, characterized by fatty and vacuolar degeneration of hepatocytes, with the presence of small, round, transparent lipid droplets and cytoplasmic vacuoles, focal hepatocellular necrosis, nuclear pyknosis, and fibrous tissue proliferation within necrotic areas. The fibrotic regions contained fibroblasts with both oval‐ and spindle‐shaped nuclei (Figure 4B and Supporting Information 15: Figure S9). According to the standardized four‐grade lesion scoring system, proliferative fibrous tissue and punctate hepatocellular necrosis were each graded as “+,” indicating involvement of 0%–10% of the examined tissue. The WT, Δ*treC*, and Δ*treC* + lysoPC groups showed minimal vacuolar and fatty degeneration of hepatocytes, with preserved hepatic architecture and no significant punctate hepatocellular necrosis, fibrous tissue proliferation, or other histopathological alterations.

These results demonstrate that lysoPC selectively amplifies *S. suis* virulence in pulmonary and hepatic tissues through a *treC*‐dependent mechanism. The pronounced genotype‐specific histopathological phenotypes implicate *treC* as a critical regulatory locus mediating lysoPC‐induced virulence.

## 4. Discussion

lysoPC is the predominant LPL in most animal tissues and is widely present in plasma and CSF of humans and animals and is also a central component in certain pathological processes [11, 26]. As a key signaling molecule in eukaryotic cells, lysoPC plays a pivotal role in regulating cell proliferation, apoptosis, the inflammatory response, and various other physiological processes [27, 28]. In contrast, the physiological function of lysoPC on bacteria is still poorly understood. Here, we elucidate a novel mechanism by which *S. suis* can hijack lysoPC from the environment to enhance virulence through metabolic reprogramming rather than classical virulence factor activation.

In this study, we showed that *S. suis* virulence can be specifically enhanced by lysoPC. In *S. suis*, lysoPC‐treated WT showed an accelerated mortality in mice and an increase in cellular LDH release, correlating with disrupted epithelial tight junctions and systemic dissemination. So far, only *Salmonella* has been shown to utilize lysoPC to reinforce virulence [8]. In this process, the release of lysoPC from host cells after infection directly promotes the expression of *Salmonella* invasion proteins (Sips) and the *Salmonella* pathogenicity island 1 (SPI‐1) to reinforce *Salmonella* invasion into the host [8, 14]. Our previous studies have shown that another phospholipid derivative, lysoPE, can enhance bacterial pathogenicity and environmental adaptability by increasing bacterial cytotoxicity and facilitating bacterial transport of iron ions, suggesting that LPLs may play an important role in the virulence evolution, pathogenicity, and transmission of pathogenic bacteria [9, 10].

Interestingly, from transcriptomic profiling we observed the absence of transcriptional changes in *S. suis* canonical virulence genes (e.g., *sly*, *mrp*, and *fbps*), despite robust lysoPC‐driven virulence enhancement. Instead, RNA‐seq revealed significant upregulation of carbohydrate metabolism, with *treC* emerging as a critical regulator. This suggests that *S. suis* prioritizes metabolic flexibility over classical virulence determinants when exposed to lysoPC. TreC is a core enzyme of bacterial energy metabolism, mainly involved in trehalose metabolism, which is absent in mammals [29, 30]. Although there have been no reports of direct regulation of bacterial virulence by TreC, *treC*‐mediated trehalose biosynthesis pathway is critical for bacterial stress tolerance, as trehalose stabilizes cellular structures under osmotic and oxidative stress [31]. Moreover, the enhancement of bacterial virulence under *treC* influence aligns with observations in other pathogenic bacteria, where trehalose and its derivatives are intricately linked to virulence. For instance, trehalose can promote colonization in the host bladder through osmoregulation and modulate the toxicity in the host and the colonization in macrophages and enhance virulence by promoting bacterial toxin secretion in *Escherichia coli*, *Burkholderia pseudomallei*, and *Clostridium difficile*, respectively [32, 33]. Other streptococcal species such as *Streptococcus mutans* and *Streptococcus pyogenes* utilize trehalose to coordinate the regulation of virulence, underscoring the critical role of trehalose metabolism in bacterial pathogenesis [32], although the exact regulatory mechanism is not yet clear. Recent studies highlight bacterial metabolic plasticity as a critical virulence strategy, where pathogens like invasive streptococci exploit host‐derived lipids to fuel stress resilience and immune evasion [34]. Our findings are consistent with this paradigm, demonstrating that lysoPC‐treated *S. suis* induces 100% murine mortality by 18 h (median survival: 15 h; *p*  < 0.0001 vs. untreated *S. suis*, Figure 1A), whereas Δ*treC* mutants exhibit no virulence enhancement (*p* = 0.36, 16.6% mortality at 48 h, Figure 3A), underscoring lysoPC enhances virulence in a *treC*‐dependent manner. Consistent with survival assay results, deletion of *treC* abolished the lysoPC‐mediated increase in bacterial virulence in both murine survival assays and cytotoxicity experiments. Genetic complementation fully restored lysoPC‐dependent pathogenicity, positioning *treC* as a central node in the lysoPC‐induced *S. suis* metabolic‐virulence connection. These findings advance our understanding of how bacterial pathogens exploit host‐derived molecules to optimize their fitness in hostile environments, aligning with the emerging paradigms that metabolic plasticity, rather than traditional virulence factors, often dictates pathogen success in hostile host environments [35].

Histopathological analysis revealed that lysoPC preferentially amplifies *S. suis* virulence in pulmonary and hepatic tissues, with minimal effects observed in the spleen and brain. This organ‐specific susceptibility and amplification of lysoPC‐enhanced virulence further underscores the interplay between bacterial metabolism and host niche dynamics and may reflect differences in lysoPC availability or tissue‐specific metabolic environments. The liver is a major site of lysoPC synthesis and metabolism in animals, which may provide an enriched niche for *S. suis* to exploit lysoPC as a metabolic signal [36]. Additionally, the pronounced hepatic and pulmonary pathology observed in lysoPC‐treated mice aligns with the clinical presentation of *S. suis* infections, which often manifest as septicemia, pneumonia, and hepatic injury in humans and animals [37, 38].

Despite these advances, key questions remain unresolved regarding how lysoPC sensing is coupled to the transcriptional upregulation of metabolic genes. Although our data establish a functional connection between lysoPC exposure and *treC*‐dependent metabolic reprogramming, the molecular mechanisms by which lysoPC is sensed and transduced into transcriptional activation of metabolic genes remain to be elucidated. One plausible mechanism is that lysoPC, as an amphipathic lipid, alters bacterial membrane composition or biophysical properties, thereby engaging membrane‐associated sensors or signal transduction systems that respond to lipid‐induced membrane perturbations. Alternatively, lysoPC may indirectly influence *treC* expression through global transcriptional regulators involved in carbon metabolism or stress adaptation, integrating lipid‐derived environmental cues with metabolic control. While these possibilities remain speculative, they provide a conceptual framework for understanding how host‐derived lipids may be coupled to bacterial metabolic reprogramming. Additionally, although murine models provide critical insights, they may not fully reproduce lysoPC dynamics or immune responses in humans. Therefore, validation in higher‐order systems is necessary.

In conclusion, this study demonstrates that lysoPC enhances *S. suis* virulence through a *treC*‐dependent metabolic reprogramming mechanism. By prioritizing trehalose metabolism, lysoPC primes *S. suis* to survive and cause damage in host tissues, particularly in the lungs and liver. These findings not only advance our understanding of *S. suis* pathogenesis but also identify *treC* as a promising target for therapeutic intervention. Targeting *treC* or its metabolic pathway could disrupt lysoPC‐mediated virulence enhancement, thereby reducing disease severity in *S. suis* infections. Furthermore, understanding how lysoPC modulates bacterial metabolism may inform strategies to manipulate host–microbe interactions for therapeutic benefit. Given the growing recognition of host metabolites as regulators of bacterial pathogenicity, our findings highlight the importance of exploring metabolic crosstalk as a novel therapeutic target. Future studies should focus on elucidating the molecular mechanisms by which lysoPC regulates *treC* expression and exploring the therapeutic potential of targeting this pathway in *S. suis* infections [39].

## Funding

This work was supported by the National Natural Science Foundation of China (Grants 32503042 and 32473027), the Fundamental Research Funds for the Central Universities (Grant SWU‐XJPY202305), the New Chongqing‐Young Elite Program (Grant CSTB2024YCJH‐KYXM0078), Natural Science Foundation of Chongqing, China (Grant CSTB2025NSCQ‐GPX0497), and the Chongqing Modern Agricultural Industry Technology System (Grant CQMAITS202512).

## Disclosure

Parts of this manuscript are based on the first author’s doctoral dissertation [39], which represents the initial documentation of this line of research. While certain similarities in wording may exist, particularly in the methodological descriptions, the present work constitutes a substantially extended of the original thesis. The manuscript includes newly generated data and updated interpretations that were not part of the doctoral dissertation. This submission represents the first formal dissemination of these findings in a peer‐reviewed scholarly publication. All authors confirm that this work complies with the journal’s policies on originality and publication ethics.

## Conflicts of Interest

The authors declare no conflicts of interest.

## Supporting Information

Additional supporting information can be found online in the Supporting Information section.

## Supporting information


**Supporting Information 1** Table S1: Gene mutagenesis primer list.

**Supporting Information 2** Table S2: Hierarchical clustering of differentially expressed genes.

**Supporting Information 3** Table S3: (A) Gene transcripts difference between *S. suis* and lysoPC‐pretreated *S. suis*. (B) The list of lysoPC‐induced upregulated genes.

**Supporting Information 4** Table S4: (A) RNA‐seq and real‐time PCR results of genes of *S. suis* regulated by lysoPC. (B) Quantitative real‐time RT‐PCR primer list.

**Supporting Information 5** Table S5: Results of KEGG enrichment analysis.

**Supporting Information 6** Table S6: Results of GO enrichment analysis.

**Supporting Information 7** Figure S1: LDH release of hCMEC/D3 cells infected with *S. suis* (A) pretreated with different LPL species or (B) exposed to increasing concentration of lysoPC.

**Supporting Information 8** Figure S2: Fluorescent microscopy images of hCMEC/D3 cells infected with *S. suis* and lysoPC‐pretreated *S. suis* at MOIs of 10 for the indicated times stained with Hoechst/PI. Propidium iodide (PI) uptake were used to determine cell death.

**Supporting Information 9** Figure S3: The heatmap and hierarchical clustering of the by lysoPC highly regulated genes in *S. suis*.

**Supporting Information 10** Figure S4: The Gene Ontology (GO) terms analysis of lysoPC‐induced transcriptome regulation.

**Supporting Information 11** Figure S5: The KEGG analysis of lysoPC‐induced transcriptome regulation.

**Supporting Information 12** Figure S6: Survival assays of mice after Δ*lacD*, Δ*treC*, lysoPC‐pretreated Δ*lacD* and lysoPC‐pretreated Δ*treC* or DPBS injection.

**Supporting Information 13** Figure S7: Growth curves of wild‐type *S. suis*, and the Δ*treC* and Δ*lacD* mutant strains in THB supplemented with lysoPC.

**Supporting Information 14** Figure S8: Fluorescent microscopy images of hCMEC/D3 cells infected with *S. suis*, Δ*treC*, lysoPC‐pretreated *S. suis*, lysoPC‐pretreated Δ*treC* infection at MOIs of 10 for the indicated times stained with Hoechst/PI. Propidium iodide (PI) uptake were used to determine cell death.

**Supporting Information 15** Figure S9: Histopathological changes in (A) lung and (B) liver of *S. suis*, Δ*treC*, lysoPC‐pretreated *S. suis*, lysoPC‐pretreated Δ*treC* infection (magnification, 100×).

## Data Availability

The authors confirm that the data supporting the findings of this study are available within the article and its Supporting Information.

## References

[1] Tram G. , Jennings M. P. , Blackall P. J. , and Atack J. M. , *Streptococcus suis* Pathogenesis-A Diverse Array of Virulence Factors for a Zoonotic Lifestyle, Advances in Microbial Physiology. (2021) 78, 217–257, 10.1016/bs.ampbs.2020.12.002. 34147186

[2] Lv R. , Zhang W. , Sun Z. , Si X. , Dong H. , and Liu X. , Current Prevalence and Therapeutic Strategies for Porcine *Streptococcus suis* in China, Applied and Environmental Microbiology. (2025) 91, no. 3, 10.1128/aem.02160-24, e0216024. 39998255 PMC11921377

[3] Dresen M. , Valentin-Weigand P. , and Berhanu Weldearegay Y. , Role of Metabolic Adaptation of *Streptococcus suis* to Host Niches in Bacterial Fitness and Virulence, Pathogens. (2023) 12, no. 4, 10.3390/pathogens12040541, 541. 37111427 PMC10144218

[4] Chugh S. , Létisse F. , and Neyrolles O. , The Exometabolome as a Hidden Driver of Bacterial Virulence and Pathogenesis, Trends in Microbiology. (2025) 33, no. 5, 546–557, 10.1016/j.tim.2024.11.009. 39701858

[5] Zhan L. , Ge J. , Xia L. , and Zhang Y. , Reciprocal Regulation Between Bacterial Secretion Systems and Host Metabolism: Enhancing Bacterial Intracellular Survival Capability, Microbiological Research. (2025) 292, 10.1016/j.micres.2024.128025, 128025. 39705830

[6] Cao X. , van Putten J. P. M. , and Wösten M. M. S. M. , *Campylobacter jejuni* Benefits From the Bile Salt Deoxycholate Under Low-Oxygen Condition in a PldA Dependent Manner, Gut Microbes. (2023) 15, no. 2, 10.1080/19490976.2023.2262592, 2262592. 37768138 PMC10540661

[7] Brizuela J. , Roodsant T. J. , and Hasnoe Q. , et al.Molecular Epidemiology of Underreported Emerging Zoonotic Pathogen *Streptococcus suis* in Europe, Emerging Infectious Diseases. (2024) 30, no. 3, 413–422, 10.3201/eid3003.230348. 38407169 PMC10902550

[8] Yuan S. , Liu B. , and Quan Y. , et al. *Streptococcus suis* Regulates Central Carbon Fluxes in Response to Environment to Balance Drug Resistance and Virulence, Microbiological Research. (2025) 296, 10.1016/j.micres.2025.128157, 128157. 40174362

[9] Cao X. , van de Lest C. H. A. , Huang L. Z. X. , van Putten J. P. M. , and Wösten M. M. S. M. , *Campylobacter jejuni* Permeabilizes the Host Cell Membrane by Short Chain Lysophosphatidylethanolamines, Gut Microbes. (2022) 14, no. 1, 10.1080/19490976.2022.2091371, 2091371. 35797141 PMC9272830

[10] Cao X. , van Putten J. P. M. , and Wösten M. M. S. M. , Biological Functions of Bacterial Lysophospholipids, Advances in Microbial Physiology. (2023) 82, 129–154, 10.1016/bs.ampbs.2022.10.001. 36948653

[11] Boldyreva L. V. , Morozova M. V. , Saydakova S. S. , and Kozhevnikova E. N. , Fat of the Gut: Epithelial Phospholipids in Inflammatory Bowel Diseases, International Journal of Molecular Sciences. (2021) 22, no. 21, 10.3390/ijms222111682, 11682. 34769112 PMC8584226

[12] Subramanian N. and Qadri A. , Lysophospholipid Sensing Triggers Secretion of Flagellin From Pathogenic *Salmonella* , Nature Immunology. (2006) 7, no. 6, 583–589, 10.1038/ni1336, 2-s2.0-33744460202. 16648855

[13] Lou L. , Zhang P. , Piao R. , and Wang Y. , *Salmonella* Pathogenicity Island 1 (SPI-1) and Its Complex Regulatory Network, Frontiers in Cellular and Infection Microbiology. (2019) 9, 10.3389/fcimb.2019.00270, 2-s2.0-85071582009, 270. 31428589 PMC6689963

[14] Shivcharan S. , Yadav J. , and Qadri A. , Host Lipid Sensing Promotes Invasion of Cells With Pathogenic *Salmonella* , Scientific Reports. (2018) 8, no. 1, 10.1038/s41598-018-33319-9, 2-s2.0-85055073110, 15501. 30341337 PMC6195605

[15] Holden M. T. , Hauser H. , and Sanders M. , et al.Rapid Evolution of Virulence and Drug Resistance in the Emerging Zoonotic Pathogen *Streptococcus suis* , PLoS ONE. (2009) 4, no. 7, 10.1371/journal.pone.0006072, 2-s2.0-67650658074. PMC2705793 19603075

[16] Shen X. , Ran J. , and Yang Q. , et al.RACK1 and NEK7 Mediate GSDMD-Dependent Macrophage Pyroptosis Upon *Streptococcus suis* Infection, Veterinary Research. (2024) 55, no. 1, 10.1186/s13567-024-01376-w, 120. 39334337 PMC11428613

[17] Takamatsu D. , Osaki M. , and Sekizaki T. , Thermosensitive Suicide Vectors for Gene Replacement in *Streptococcus suis* , Plasmid. (2001) 46, no. 2, 140–148, 10.1006/plas.2001.1532, 2-s2.0-0034781075. 11591139

[18] Chen T. , Huang Q. , Li Z. , Zhang W. , Lu C. , and Yao H. , Construction and Characterization of a *Streptococcus suis* Serotype 2 Recombinant Expressing Enhanced Green Fluorescent Protein, PLoS ONE. (2012) 7, no. 7, 10.1371/journal.pone.0039697, 2-s2.0-84864089430, e39697. 22911688 PMC3401235

[19] Takamatsu D. , Osaki M. , and Sekizaki T. , Construction and Characterization of *Streptococcus suis*-*Escherichia coli* Shuttle Cloning Vectors, Plasmid. (2001) 45, no. 2, 101–113, 10.1006/plas.2000.1510, 2-s2.0-0035099359. 11322824

[20] Auger J. P. , Christodoulides M. , Segura M. , Xu J. , and Gottschalk M. , Interactions of *Streptococcus suis* Serotype 2 With Human Meningeal Cells and Astrocytes, BMC Research Notes. (2015) 8, no. 1, 10.1186/s13104-015-1581-2, 2-s2.0-84945268774, 607. 26502903 PMC4624383

[21] Cao X. , Brouwers J. , and van Dijk L. , et al.The Unique Phospholipidome of the Enteric Pathogen *Campylobacter jejuni*: Lysophosholipids Are Required for Motility at Low Oxygen Availability, Journal of Molecular Biology. (2020) 432, no. 19, 5244–5258, 10.1016/j.jmb.2020.07.012. 32710984

[22] Cao X. , Jia K. , and Liu Q. , et al.The Critical Role of NLRP3 Inflammasome Activation in *Streptococcus suis*-Induced Blood-Brain Barrier Disruption, Veterinary Microbiology. (2024) 295, 10.1016/j.vetmic.2024.110161, 110161. 38945021

[23] Matute-Bello G. , Downey G. , and Moore B. B. , et al.An Official American Thoracic Society Workshop Report: Features and Measurements of Experimental Acute Lung Injury in Animals, American Journal of Respiratory Cell and Molecular Biology. (2011) 44, no. 5, 725–738, 10.1165/rcmb.2009-0210ST, 2-s2.0-79955487598. 21531958 PMC7328339

[24] Goodman Z. D. , Grading and Staging Systems for Inflammation and Fibrosis in Chronic Liver Diseases, Journal of Hepatology. (2007) 47, no. 4, 598–607, 10.1016/j.jhep.2007.07.006, 2-s2.0-34548277150. 17692984

[25] Gibson-Corley K. N. , Olivier A. K. , and Meyerholz D. K. , Principles for Valid Histopathologic Scoring in Research, Veterinary Pathology. (2013) 50, no. 6, 1007–1015, 10.1177/0300985813485099, 2-s2.0-84887527334. 23558974 PMC3795863

[26] Hayakawa K. , Kurano M. , and Ohya J. , et al.Lysophosphatidic Acids and Their Substrate Lysophospholipids in Cerebrospinal Fluid as Objective Biomarkers for Evaluating the Severity of Lumbar Spinal Stenosis, Scientific Reports. (2019) 9, no. 1, 10.1038/s41598-019-45742-7, 2-s2.0-85067894052, 9144. 31235770 PMC6591408

[27] Prabutzki P. , Schiller J. , and Engel K. M. , Phospholipid-Derived Lysophospholipids in (patho)physiology, Atherosclerosis. (2024) 398, 10.1016/j.atherosclerosis.2024.118569, 118569. 39227208

[28] Jose A. , Fernando J. J. , and Kienesberger P. C. , Lysophosphatidic Acid Metabolism and Signaling in Heart Disease, Canadian Journal of Physiology and Pharmacology. (2024) 102, no. 12, 685–696, 10.1139/cjpp-2024-0077. 38968609

[29] Perfect J. R. , Tenor J. L. , Miao Y. , and Brennan R. G. , Trehalose Pathway as an Antifungal Target, Virulence. (2016) 8, no. 2, 143–149, 10.1080/21505594.2016.1195529, 2-s2.0-84975152218. 27248439 PMC5383216

[30] Ruhal R. , Kataria R. , and Choudhury B. , Trends in Bacterial Trehalose Metabolism and Significant Nodes of Metabolic Pathway in the Direction of Trehalose Accumulation, Microbial Biotechnology. (2013) 6, no. 5, 493–502, 10.1111/1751-7915.12029, 2-s2.0-84882283752. 23302511 PMC3918152

[31] Wu J. , McAuliffe O. , and O’Byrne C. P. , Trehalose Transport Occurs via TreB in *Listeria monocytogenes* and It Influences Biofilm Development and Acid Resistance, International Journal of Food Microbiology. (2023) 394, 10.1016/j.ijfoodmicro.2023.110165, 110165. 36933360

[32] Vanaporn M. and Titball R. W. , Trehalose and Bacterial Virulence, Virulence. (2020) 11, no. 1, 1192–1202, 10.1080/21505594.2020.1809326. 32862781 PMC7549927

[33] Klemberg V. S. , Pavanelo D. B. , and Houle S. , et al.The Osmoregulated Metabolism of Trehalose Contributes to Production of Type 1 Fimbriae and Bladder Colonization by Extraintestinal *Escherichia coli* Strain BEN2908, Frontiers in Cellular and Infection Microbiology. (2024) 14, 10.3389/fcimb.2024.1414188, 1414188. 38979511 PMC11228248

[34] Bhagwat A. , Haldar T. , Kanojiya P. , and Saroj S. D. , Bacterial Metabolism in the Host and Its Association With Virulence, Virulence. (2025) 16, no. 1, 10.1080/21505594.2025.2459336, 2459336. 39890585 PMC11792850

[35] Guo X. , Ji N. , and Guo Q. , et al.Metabolic Plasticity, Essentiality and Therapeutic Potential of Ribose-5-Phosphate Synthesis in Toxoplasma Gondii, Nature Communications. (2024) 15, no. 1, 10.1038/s41467-024-47097-8, 2999. PMC11001932 38589375

[36] Harada S. , Taketomi Y. , and Aiba T. , et al.The Lysophospholipase PNPLA7 Controls Hepatic Choline and Methionine Metabolism, Biomolecules. (2023) 13, no. 3, 10.3390/biom13030471, 471. 36979406 PMC10046082

[37] Hu X. , Lu Y. , Yu X. , Jia K. , Xiong Q. , and Fang R. , The Suppressive Role of NLRP6 in Host Defense Against *Streptococcus suis* Infection, Veterinary Microbiology. (2024) 296, 10.1016/j.vetmic.2024.110166, 110166. 38968694

[38] Liedel C. , Rieckmann K. , and Baums C. G. , A Critical Review on Experimental *Streptococcus suis* Infection in Pigs With a Focus on Clinical Monitoring and Refinement Strategies, BMC Veterinary Research. (2023) 19, no. 1, 10.1186/s12917-023-03735-9, 188. 37798634 PMC10552360

[39] Cao X. , Lysophospholipids - Underestimated Molecules of the Unique Phospholipidome of Campylobacter jejuni (Doctoral Dissertation), 2022, Utrecht University.

