# Supplementary figures and images for: TreC: A Critical Mediator of lysoPC‐Induced Hypervirulence in Streptococcus suis
Source: Transbound Emerg Dis. 2026 Apr 18;2026:8378013. doi: 10.1155/tbed/8378013 (PMC13091233; doi:10.1155/tbed/8378013)

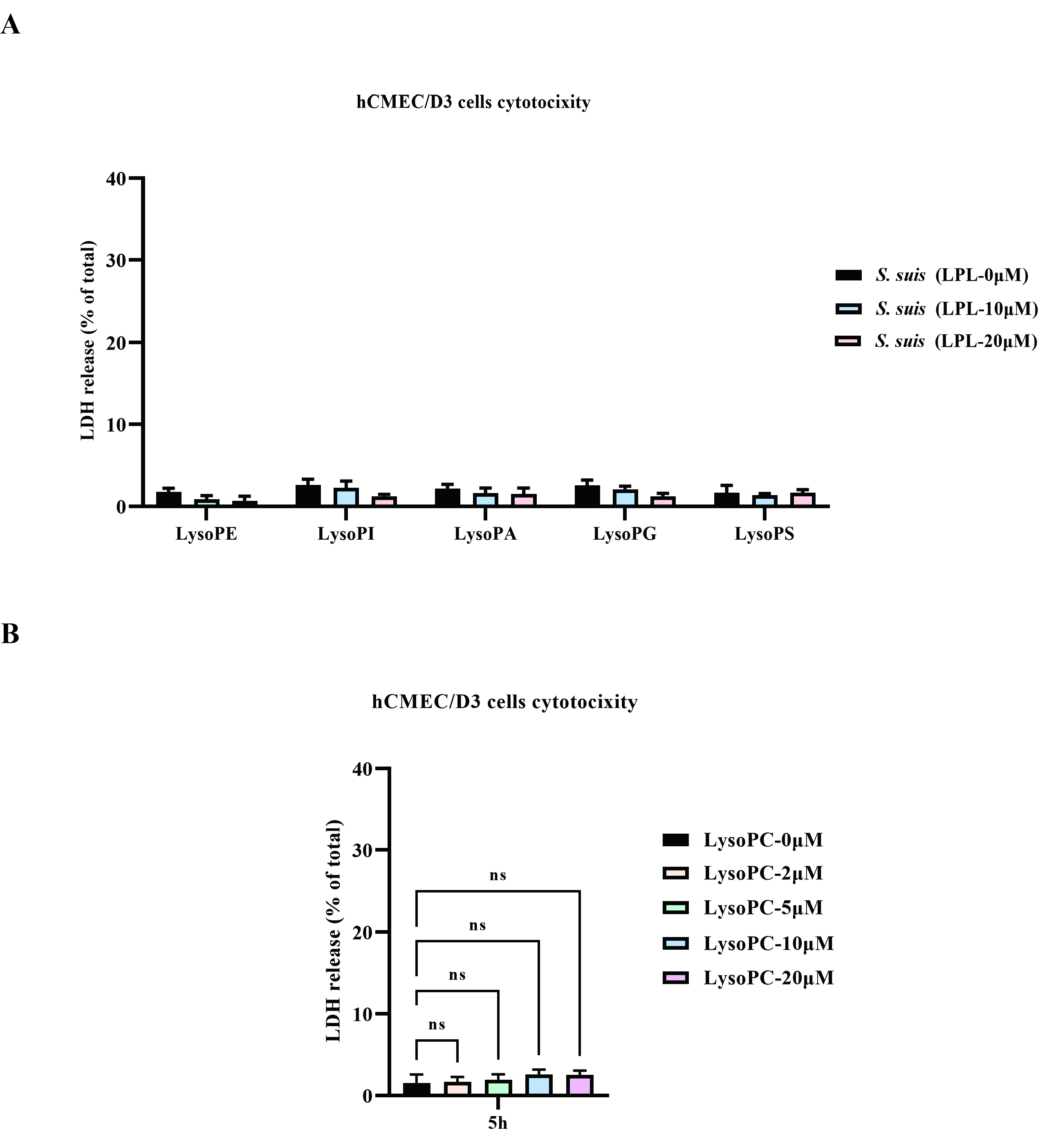

Supplement: Supplementary file 7 — Supporting Information 7 Figure S1: LDH release of hCMEC/D3 cells infected with S. suis (A) pretreated with different LPL species or (B) exposed to increasing concentration of lysoPC. [file TBED-2026-8378013-s006.docx]

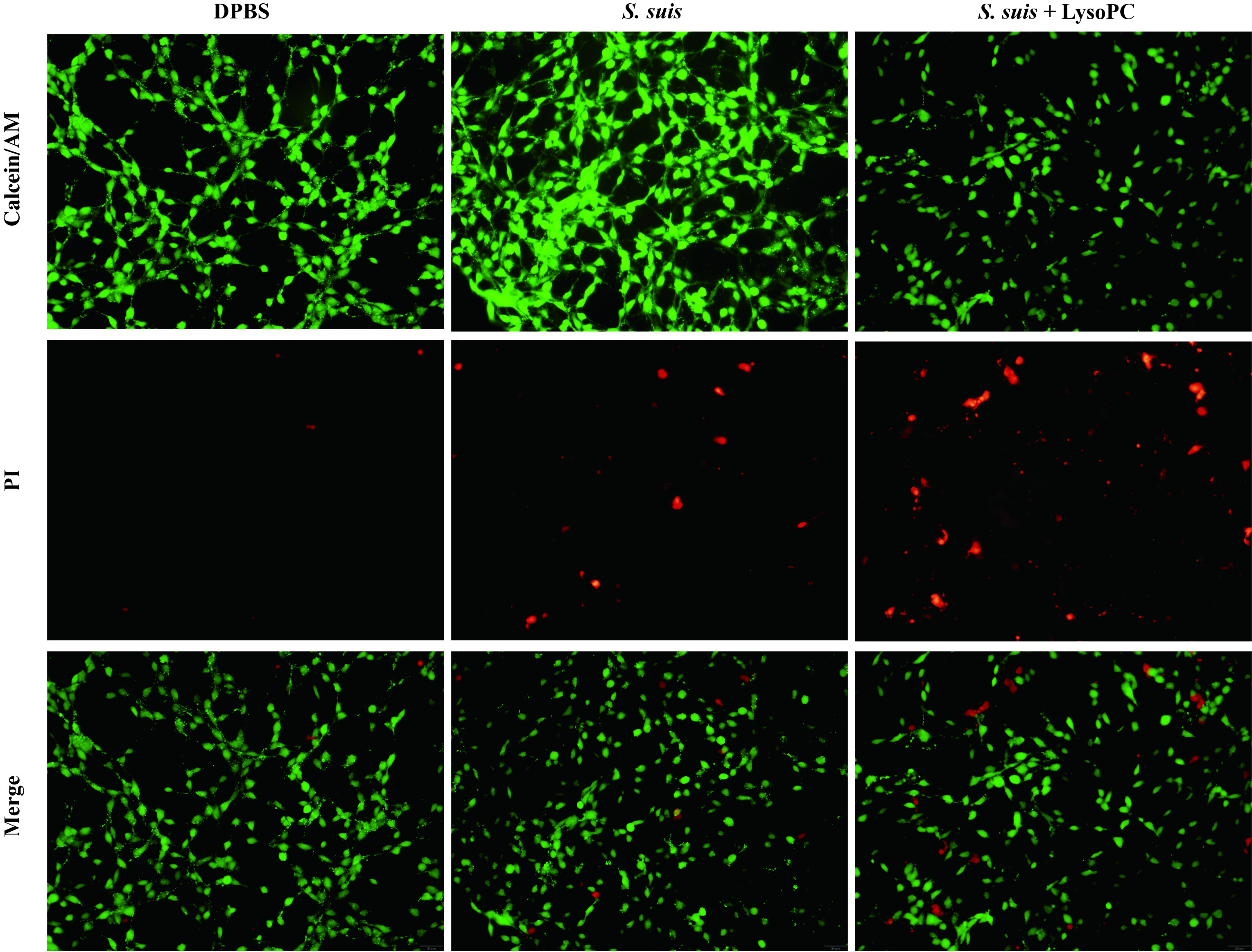

Supplement: Supplementary file 8 — Supporting Information 8 Figure S2: Fluorescent microscopy images of hCMEC/D3 cells infected with S. suis and lysoPC‐pretreated S. suis at MOIs of 10 for the indicated times stained with Hoechst/PI. Propidium iodide (PI) uptake were used to determine cell death. [file TBED-2026-8378013-s007.docx]

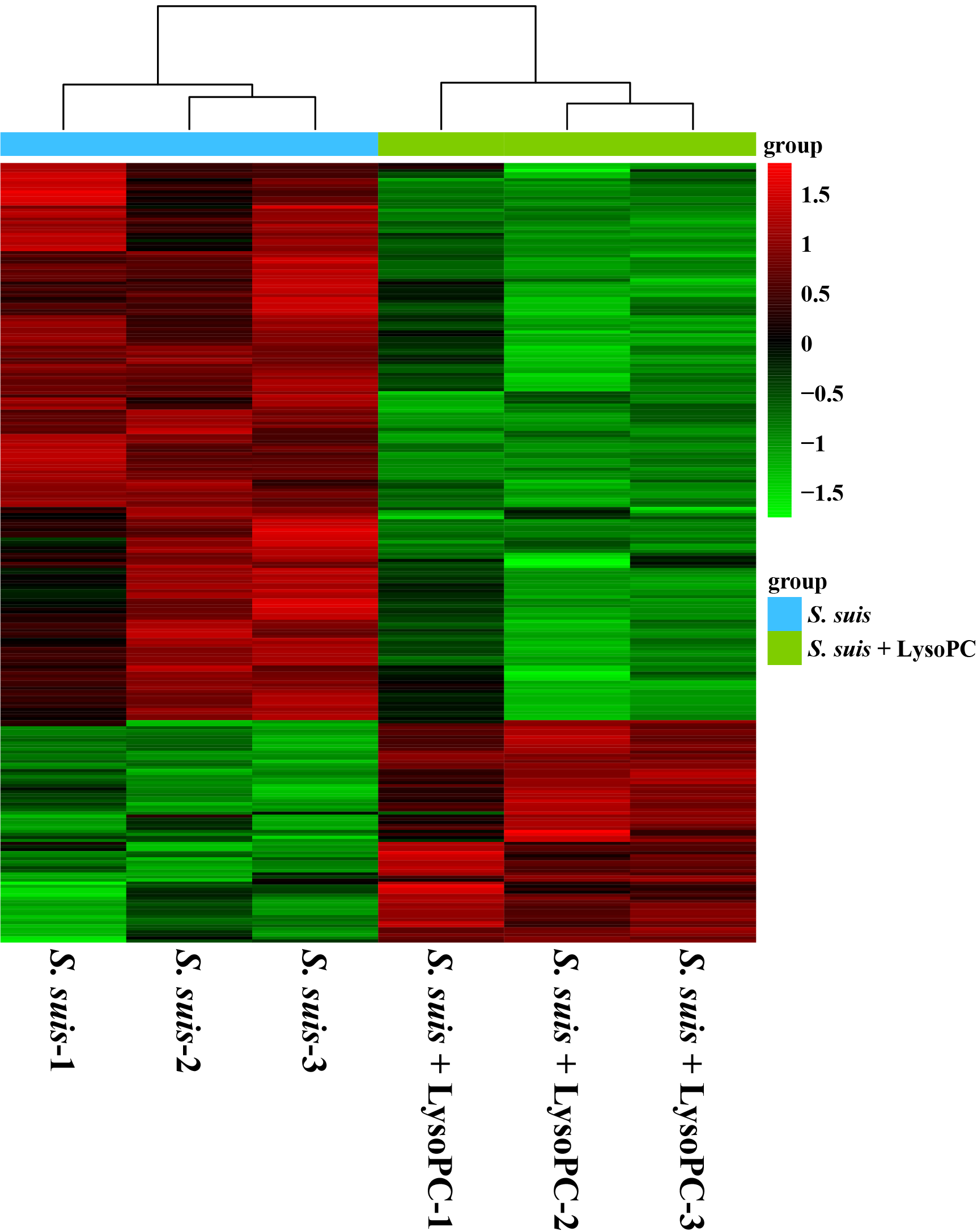

Supplement: Supplementary file 9 — Supporting Information 9 Figure S3: The heatmap and hierarchical clustering of the by lysoPC highly regulated genes in S. suis. [file TBED-2026-8378013-s008.docx]

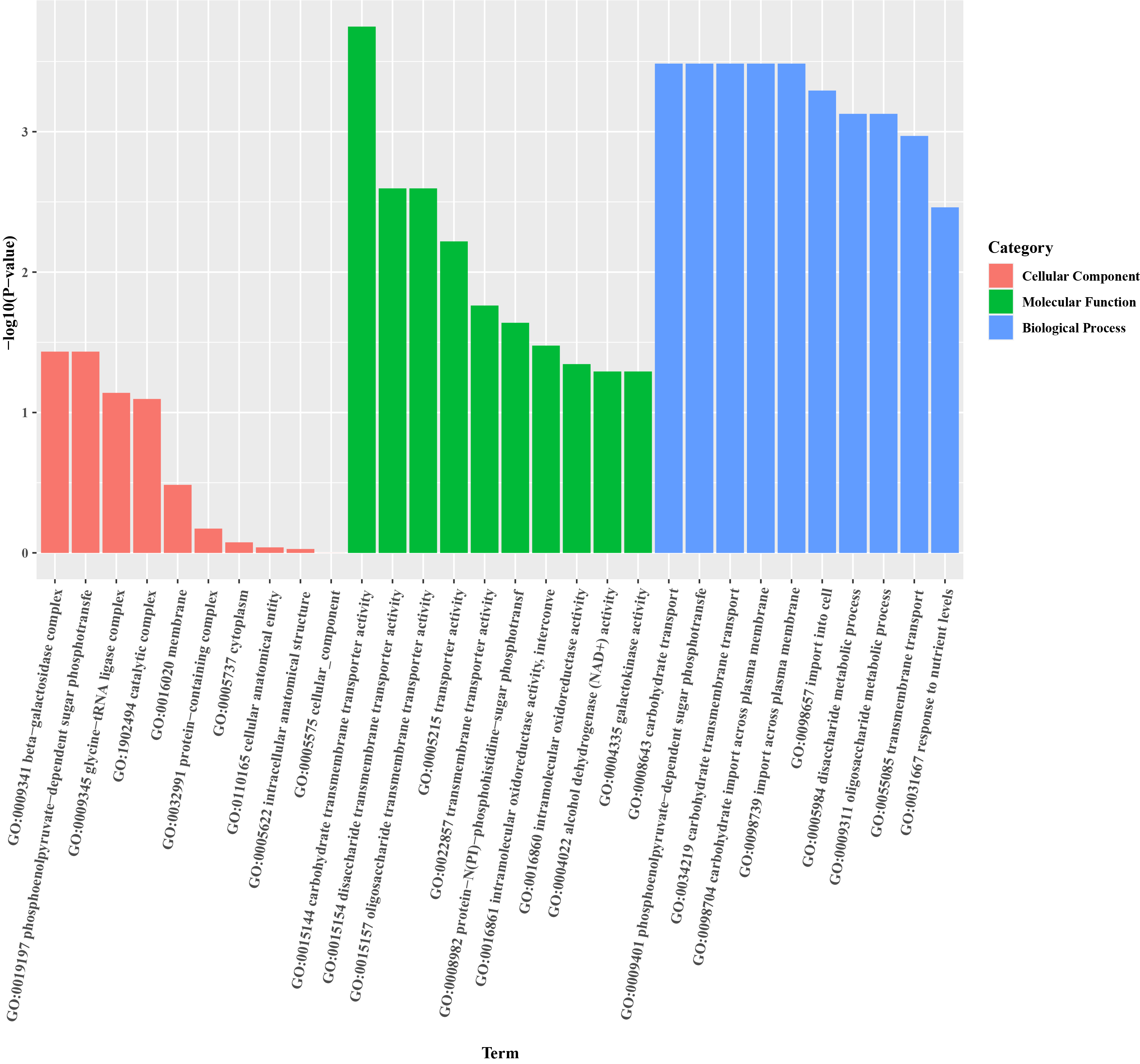

Supplement: Supplementary file 10 — Supporting Information 10 Figure S4: The Gene Ontology (GO) terms analysis of lysoPC‐induced transcriptome regulation. [file TBED-2026-8378013-s009.docx]

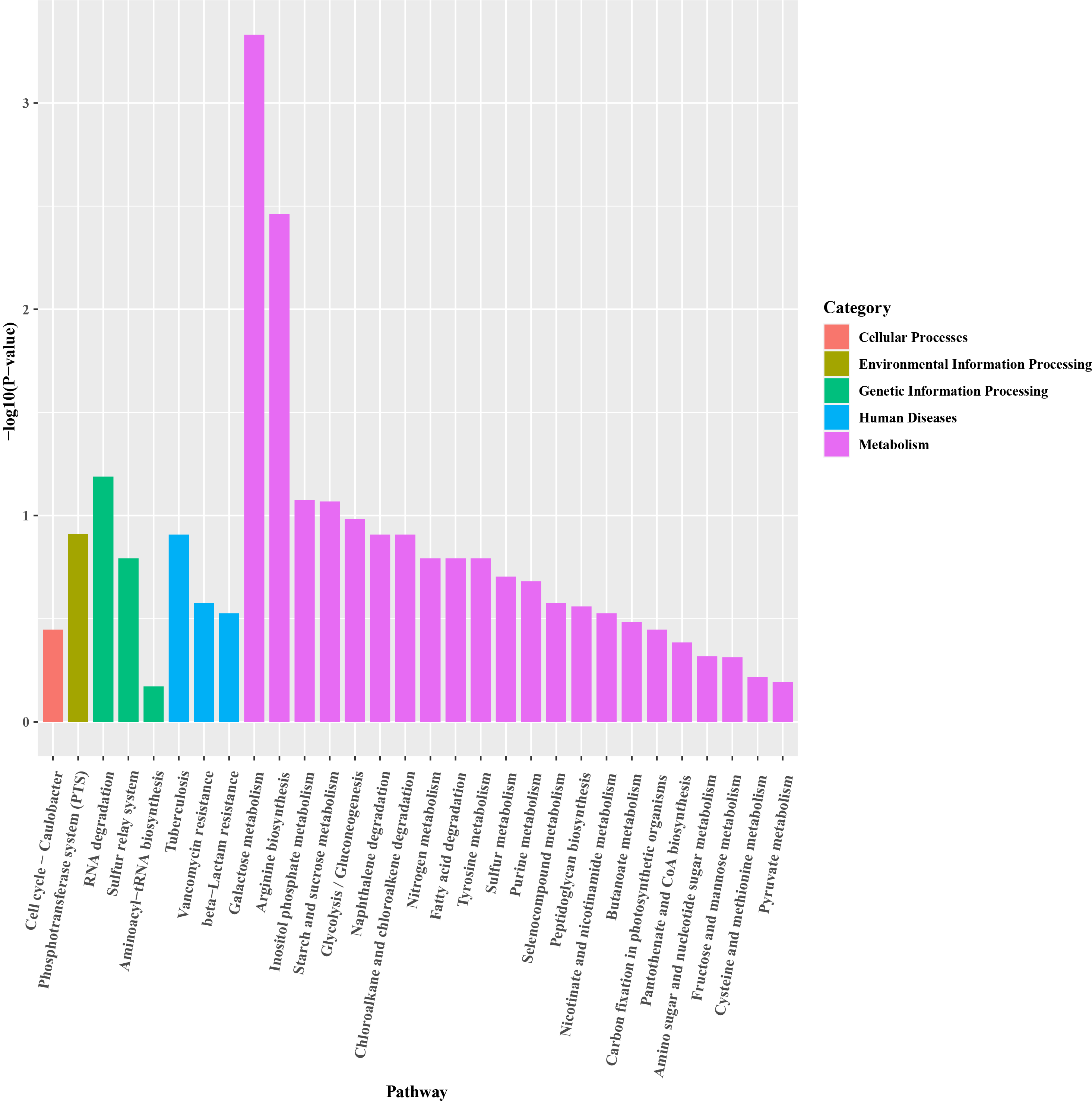

Supplement: Supplementary file 11 — Supporting Information 11 Figure S5: The KEGG analysis of lysoPC‐induced transcriptome regulation. [file TBED-2026-8378013-s010.docx]

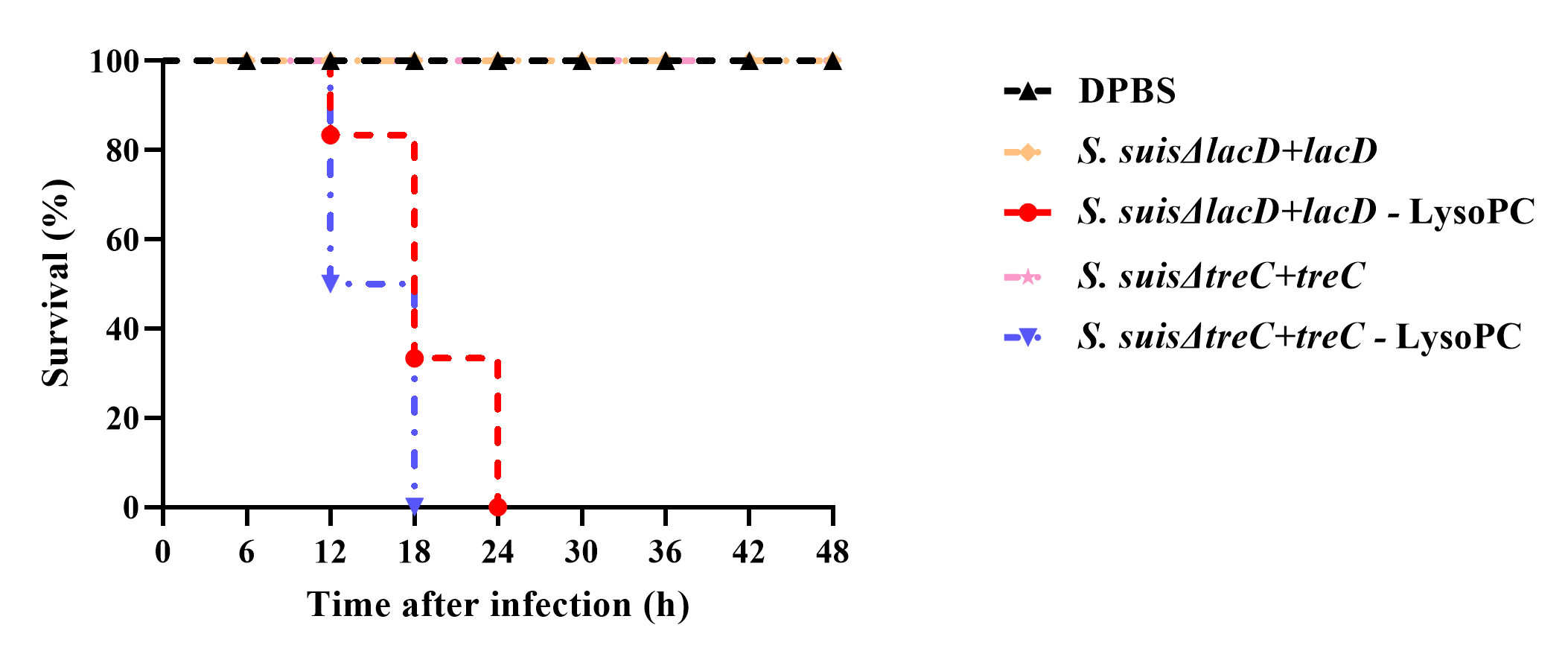

Supplement: Supplementary file 12 — Supporting Information 12 Figure S6: Survival assays of mice after ΔlacD, ΔtreC, lysoPC‐pretreated ΔlacD and lysoPC‐pretreated ΔtreC or DPBS injection. [file TBED-2026-8378013-s011.docx]

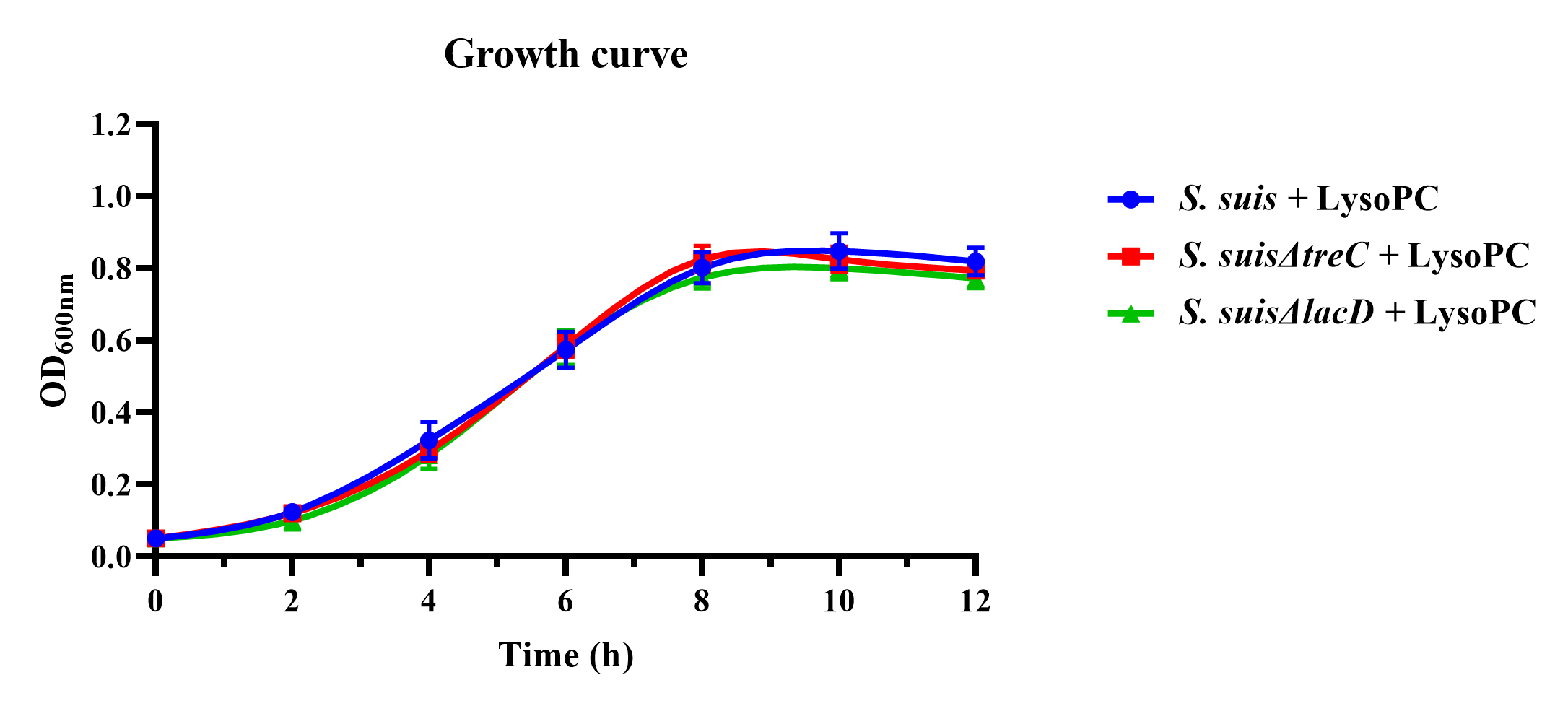

Supplement: Supplementary file 13 — Supporting Information 13 Figure S7: Growth curves of wild‐type S. suis, and the ΔtreC and ΔlacD mutant strains in THB supplemented with lysoPC. [file TBED-2026-8378013-s012.docx]

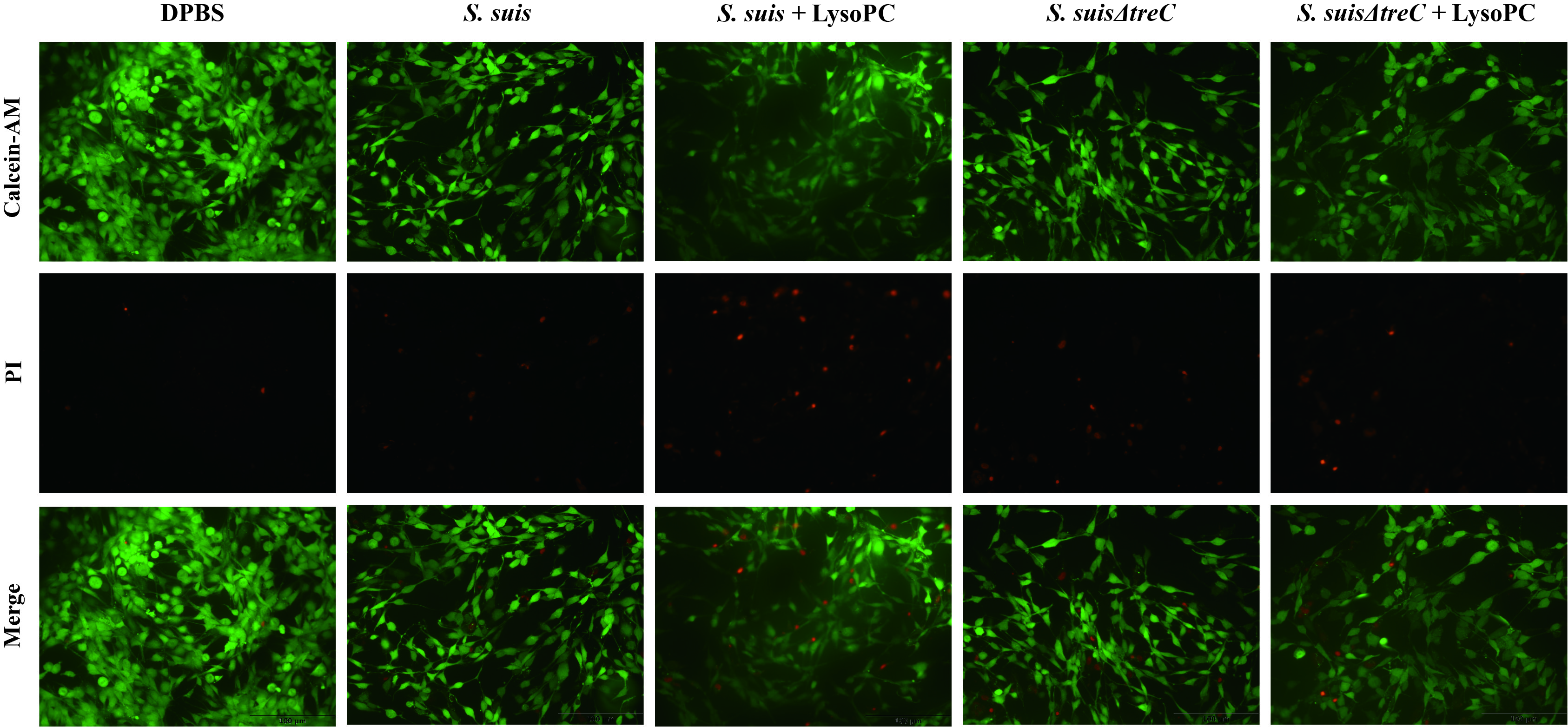

Supplement: Supplementary file 14 — Supporting Information 14 Figure S8: Fluorescent microscopy images of hCMEC/D3 cells infected with S. suis, ΔtreC, lysoPC‐pretreated S. suis, lysoPC‐pretreated ΔtreC infection at MOIs of 10 for the indicated times stained with Hoechst/PI. Propidium iodide (PI) uptake were used to determine cell death. [file TBED-2026-8378013-s013.docx]
