# Supplementary material for: TreC: A Critical Mediator of lysoPC‐Induced Hypervirulence in Streptococcus suis
Source: Transbound Emerg Dis. 2026 Apr 18;2026:8378013. doi: 10.1155/tbed/8378013 (PMC13091233; doi:10.1155/tbed/8378013)
Supplement: Supplementary file 8 [file TBED-2026-8378013-s014.docx]

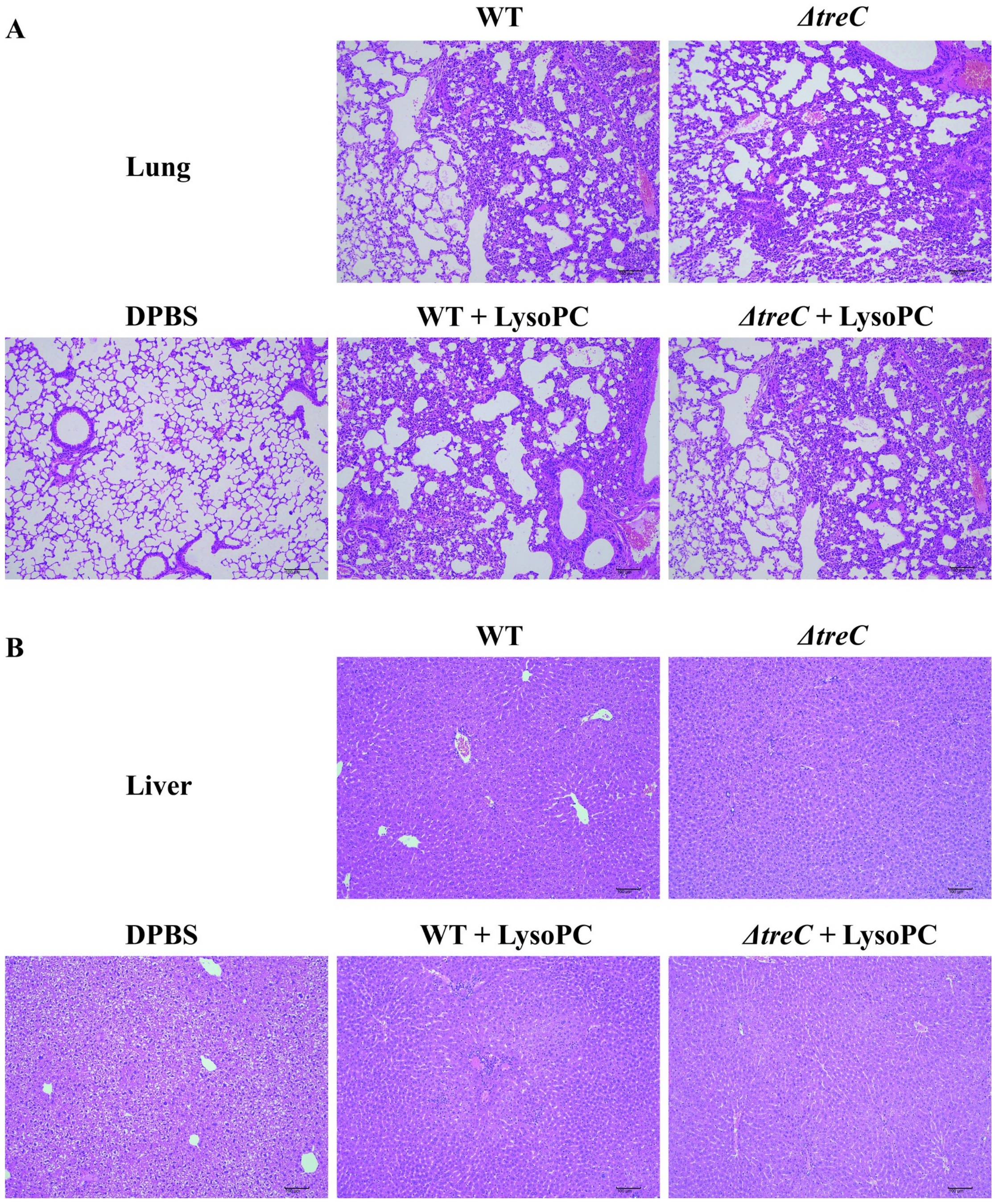
A
WT
ΔtreC
Lung
DPBS
WT + LysoPC
ΔtreC + LysoPC
B
WT
ΔtreC
Liver
DPBS
WT + LysoPC
ΔtreC + LysoPC
